# Spatiotemporal Fluctuations and Triggers of Ebola Virus Spillover

**DOI:** 10.3201/eid2303.160101

**Published:** 2017-03

**Authors:** John Paul Schmidt, Andrew W. Park, Andrew M. Kramer, Barbara A. Han, Laura W. Alexander, John M. Drake

**Affiliations:** University of Georgia Odum School of Ecology, Athens, Georgia, USA (J.P. Schmidt, A.W. Park, A.M. Kramer, J.M. Drake);; University of Georgia Center for the Ecology of Infectious Diseases, Athens (J.P. Schmidt, A.W. Park, A.M. Kramer, J.M. Drake);; Cary Institute of Ecosystem Studies, Millbrook, New York, USA (B.A. Han);; University of California–Berkeley Department of Ecology, Berkeley, California, USA (L.W. Alexander)

**Keywords:** early warning system, Ebola virus, spatiotemporal forecasting, seasonality, disease outbreaks, spillovers, infectious diseases, viruses, modeling

## Abstract

Because the natural reservoir of Ebola virus remains unclear and disease outbreaks in humans have occurred only sporadically over a large region, forecasting when and where Ebola spillovers are most likely to occur constitutes a continuing and urgent public health challenge. We developed a statistical modeling approach that associates 37 human or great ape Ebola spillovers since 1982 with spatiotemporally dynamic covariates including vegetative cover, human population size, and absolute and relative rainfall over 3 decades across sub-Saharan Africa. Our model (area under the curve 0.80 on test data) shows that spillover intensity is highest during transitions between wet and dry seasons; overall, high seasonal intensity occurs over much of tropical Africa; and spillover intensity is greatest at high (>1,000/km^2^) and very low (<100/km^2^) human population densities compared with intermediate levels. These results suggest strong seasonality in Ebola spillover from wild reservoirs and indicate particular times and regions for targeted surveillance.

Emerging infectious diseases, a persistent threat to global public health, are often linked to rapid environmental change and increasing human mobility (*1*,*2*). Notable for its unprecedented size and geographic extent, the 2013–2015 West Africa Ebola epidemic was also the first major human Ebola outbreak outside central Africa and underscored the need for improved methods to forecast emergence in novel regions. Because the natural reservoir of the Ebola virus has not been identified (*3*) and spillovers present an irregular pattern (*4*,*5*), it remains unclear how the probability of Ebola virus disease (EVD) in human populations varies in space and time. Particularly, whether EVD follows a seasonal pattern (*6*,*7*) and which historically unaffected geographic regions may also be at risk for EVD outbreaks (*8*) are 2 important questions that remain largely unanswered. Likewise, how expanding human activities, changing settlement patterns, and increasing population density affect the probability of spillovers remains poorly resolved. Despite the absence of an obvious explanation for the timing and location of past EVD outbreaks, a set of associated social and environmental conditions that anticipate viral spillover may be broadly identifiable. Identifying the environmental correlates that bring us closer to forecasting when and where EVD risk is elevated is critical for improving surveillance and rapid response to future spillovers.

Research on Ebola during the past 2 decades has investigated spatiotemporal disease probability by using conventional time series analysis (*9*) and geostatistical models (*10*). By using time series of satellite imagery, multiple studies have suggested that Ebola spillover to humans is more likely to occur at the onset of the dry season (*7*,*11*,*12*). Noting that this pattern is not universal, Lash et al. (*13*) analyzed patterns in the time series of vegetation greenness and land surface moisture (by using a normalized difference vegetation index) for 5 spillover events and found anomalies (i.e., extreme climatic fluctuations) at a temporal scale of 20 days preceding this subset of spillover events. More recently, species distribution models have been used to map the potential geographic extent of disease probability, as in the work of Pigott et al. (*4*), who used these models to identify spatial covariates that associate with the occurrence of Ebola virus infection in humans, primates, and bats.

Despite these advances, notable technology gaps remain. For example, we know that spatiotemporal variation and seasonality are key characteristics of EVD regions, but we lack integrative models that reliably incorporate spatiotemporally varying indicators of interannual and intraannual fluctuations into the calculation of spillover probability. Further, although socioeconomic factors are believed to be important drivers of spillover for numerous zoonotic diseases, including Ebola (*14*,*15*), the relationship between human population growth and the increasing frequency of EVD outbreaks since the early 1990s remains largely unexplored. We also know that the biology of this region is strongly influenced by climatic seasonality (e.g., the timing of fruit and forage availability and animal migrations). Although such seasonality is widely suspected to affect viral amplification and transmission from wild reservoirs, time-series of climate or vegetation have not been investigated across the region of documented EVD events. In this study, we combine spatial data on changing human population density and distribution during the past 4 decades, satellite-derived estimates of monthly rainfall for most of the same period, and summary measures of climate in a statistical model that dynamically captures the timing of past EVD events. Our model predicts human EVD outbreaks with an estimated accuracy of 80% and shows how EVD risk shifts seasonally as a function of environmental triggers and has varied over the last 3 decades because of increases in human population and changing settlement patterns.

## Methods

### Ebola Spillover Origin Points and Dates

We compiled a table of all known Ebola epizootics and human outbreaks from primary sources and filtered the entries to isolate primary dates and precise locations of distinct spillover events. For human Ebola spillovers, we began with chronological lists compiled by the World Health Organization and the Centers for Disease Control and Prevention. Key sources were Lahm et al. (*16*) and Leroy et al. (*17*), who compiled reports of wildlife mortality in Gabon and the Democratic Republic of the Congo; reports by ethnologists observing great ape populations in other regions; coordinates of locations from Mylne et al. (*18*); and locations and associated information from Kuhn’s compendium (*5*).

**Figure 1:**
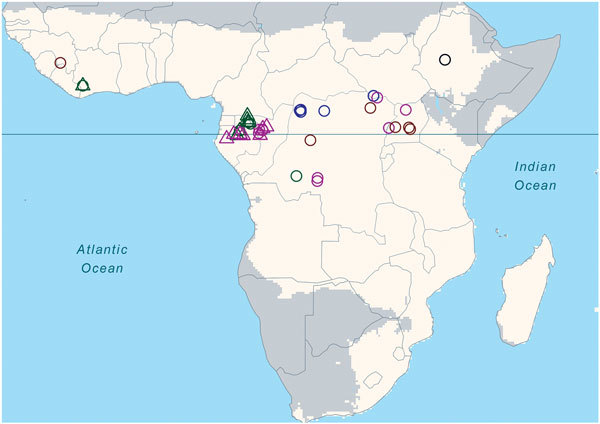
Locations of known Ebola virus spillover events, Africa, 1960–2010. Light-shaded area indicates the focal region in Africa of annual rainfall >500 mm. Open circles indicate human spillovers, open triangles infection/mortality in nonhuman primates or in other mammals. Yellow, blue, green, magenta, and black indicate the 5 respective decades during 1960–2010. Solid horizontal line marks the equator. No known Ebola spillovers occurred in the 1980s.

To divide incident reports into discrete spillover events, we separated primary spillovers from secondary occurrences on the basis of widely accepted chronological, geographic, or genetic distances. For example, where viral sequence data indicated that multiple spillover events had occurred, we considered them as such even if they overlapped spatially or temporally. Most events were reported as points. When events were reported as polygons (3 cases), we used polygon centroids as point locations. In contrast to Pigott et al. (*4*), we excluded data derived from sampling of healthy bats not associated with a spillover event. Because we were seeking to identify potential climatologic triggers, the timing of the spillover was taken to be the earliest report (often unconfirmed) of either human or animal disease rather than the first date of confirmed infection in either humans or animals. Following this procedure, a primary list of 66 spatiotemporal candidate spillover points was reduced to a final list of 44 spillover events (Figure 1; Technical Appendix 1).

### Spatial Predictors

To exclude arid and semi-arid regions, which are unlikely to harbor potential Ebola reservoir species and differ sharply in climate from locations where human EVD has occurred, we defined the region of interest as the portion of Africa receiving >500 mm rainfall annually. For this region, we assembled spatial data that capture the major sources of variation in climate and landcover. Following Pigott et al. (*4*), we chose an enhanced vegetation index (*19*) and potential evapotranspiration (*20*) to represent composite axes of coarse environmental variation.

### Candidate Triggers

To characterize spatiotemporal variation at seasonal, interannual, and decadal scales, we compiled 3 datasets. First, we compiled population count grids for Africa for 1960, 1970, 1980, 2000, 2005, 2010, and 2015 at 2.5 arc-minutes scale (≈25 km^2^ at the equator) from the Gridded Population of the World version 3, produced by the Columbia University Center for International Earth Science Information Network (http://sedac.ciesin.columbia.edu/data/collection/gpw-v3). After linearly interpolating counts by grid cell for intervening years, we log_10_-transformed values of human population and created 3 population bins according to *x*<10^2^, 10^2^<*x*<10^3^, and *x*>10^3^.

Second, we aggregated monthly rainfall from daily rainfall estimates obtained from the Rainfall Estimator (*21*). This data product was developed in 1998 by the Climate Prediction Center at the National Oceanic and Atmospheric Administration and is available at high (0.1°) spatial resolution for January 1983 to the present.

Third, in addition to actual monthly rainfall, we created a rainfall anomaly index as a means of incorporating the potential importance of relative rainfall. For the time series of 384 monthly rainfall rasters, we divided the value of each month-location by the maximum value for that location to create a set of 384 scaled raster images corresponding to the original monthly rainfall raster images.

### Model Fitting and Validation

We restricted analysis to the 37 (80.5%) of 44 EVD events occurring since 1982, the period for which monthly rainfall estimates across Africa were available. We sampled 100,000 random background points from within the portion of Africa receiving >500 mm rainfall annually and randomly assigned each to 1 of 384 months during 1983–2014. Spillover occurrence points and background points were divided into 2/3 training and 1/3 test sets. Because actual monthly rainfall at month and site of EVD outbreak varied considerably, we stratified by rainfall, first ranking points by rainfall amount and then assigning every third point to the test set.

### Model Testing

We modeled Ebola spillover intensity, the average density or expected number of points per unit area and time, using bagged logistic regression models with main effects only (*22*). Bagging (bootstrap aggregating) is a machine learning approach that uses the predictive power generated from ensembles of models based on small subsets of the data (*23*). By using all 5 predictors described, we fit 1,000 models in which we randomly sampled 10 of the 22 outbreaks in the training dataset and 100 of 100,000 training background points. We predicted each of the 1,000 fitted models on both the training and test datasets. Taking the mean of predicted spillover intensity across model iterations, we compared average predicted spillover intensity for training and test points with labels at each point (known EVD event vs. otherwise) in each dataset to gauge the accuracy of the models.

### Risk Mapping

The set of known EVD events represent a spatiotemporal point process. Point processes are described by an intensity function (i.e., the average density or expected number of points per unit area and time). Therefore, after validation, we used the complete dataset (37 spillover and 100,000 background points) to retroactively predict Ebola spillover intensity across the entire portion of Africa receiving >500 mm rainfall annually for all 384 months for which gridded rainfall data were available (January 1983–December 2014) using human population estimates for 2015. We then averaged the resulting 384 monthly rasters to map seasonal shifts in predicted Ebola spillover intensity across Africa. To map the change in spillover intensity as a function of changes in human population size and distribution across 4 decades, we averaged predicted intensity across all months of 1975 and 2015, then took the difference between annual spillover intensity in 2015 and annual spillover intensity in 1975 across the region of Africa receiving >500 mm rainfall annually.

Detailed methods are provided in Technical Appendix 2, and the R code used is provided in Technical Appendix 3. All data and code are available online (https://figshare.com/articles/ebola_spillover_intensity_final_Rmd/4234280).

## Results

**Figure 2:**
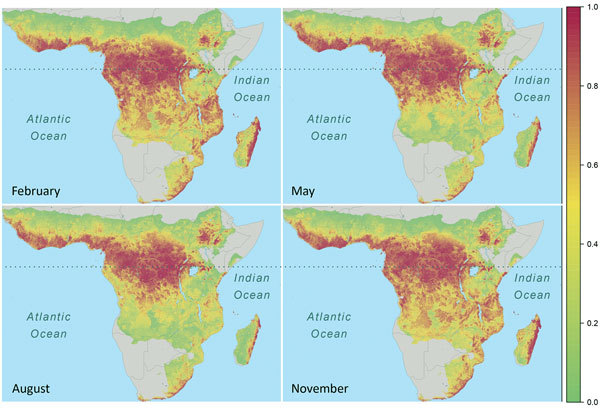
Seasonal spatiotemporal dynamics of Ebola virus spillover intensity (i.e., average density or expected number of points per unit area and month) as percentile values ranking predicted intensities at all grid cell locations within the region of Africa where annual rainfall was >500 mm for all months from January 1983 through December 2014. Panels capture shifts in the geographic pattern of spillover intensity seasonally. Dotted horizontal line marks the equator.

**Figure 3:**
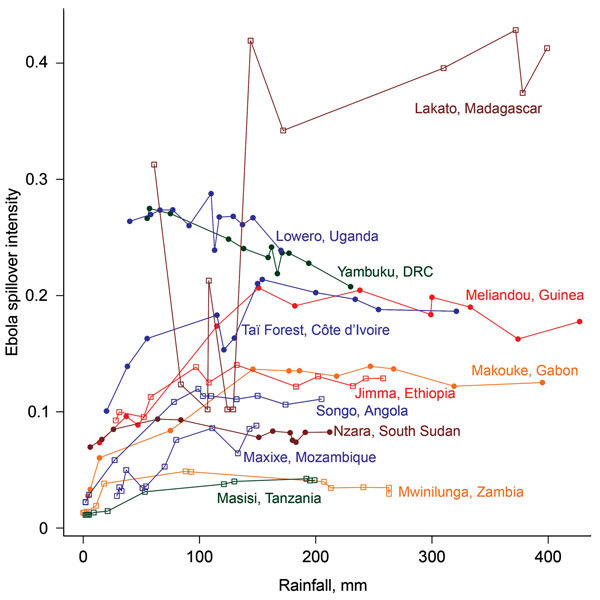
Phase graph showing the relationship between mean monthly rainfall and raw Ebola spillover intensity (defined as average density or expected number of points per unit area and/or time) for known Ebola virus disease locations in West and Central Africa (closed circles) and locations in northeastern or southern Africa where model results indicate moderate to high Ebola spillover intensity seasonally (open squares). Points are ordered by least to greatest monthly rainfall at each site. Dotted horizontal line marks the equator. DRC, Democratic Republic of the Congo.

Predictive accuracy of the bagged model of EVD intensity trained on the 2/3 training dataset was high. Area under the receiver-operator curve was 0.83 when evaluated on the training dataset and 0.80 when evaluated on 1/3 of the data that were withheld from model training. Overall accuracy ([true positives + true negatives] / total points) was 53% for prediction on the test set. Mean annual Ebola spillover intensity was highest where the enhanced vegetation index is highest in the wettest portions of tropical Africa. For locations within the humid tropics of Africa, predicted spillover intensity was generally, but not always, lowest in dry months (rainfall <50 mm) (Figure 2; Video). Across sites, modeled spillover intensity in months of intermediate rainfall (100–250 mm) was equal to or exceeded that in high (>250 mm) rainfall months (Figure 3). Whereas central Africa exhibits relatively constant spillover intensity throughout the year (particularly within the narrow equatorial region of 15°–30° longitude), we found spillover intensity to be highly seasonal in southern Africa and somewhat variable in West Africa. These results extend the proposed potential range of spillover far beyond the locations of past outbreaks. Compared with previously published spatial models of spillover that did not include temporally varying predictors, our results suggest that a much larger area of Africa is at moderate to high risk for spillover during some months of the year, including much of East Africa, Madagascar, and south central Africa (e.g., Angola and Zambia) and a large portion of West Africa (online video). Although Ebola spillover intensity in seasonally at-risk regions peripheral to central Africa is much lower than in high-intensity central Africa itself, predicted spillover intensity at sites in Angola, Mozambique, and Ethiopia is comparable to that predicted at known spillover locations in South Sudan and Gabon. Predicted spillover intensity over a large portion of Madagascar is similar to that of central Africa (Figure 3). Thus, within the African tropics, the potential for Ebola spillover appears to be geographically widespread.

**Video:**
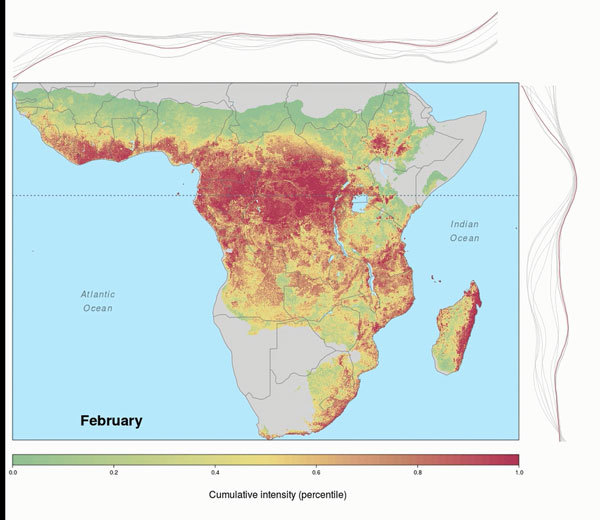
Seasonal spatiotemporal dynamics of Ebola virus spillover intensity (i.e., average density or expected number of points per unit area and month) as percentile values ranking predicted intensities at all grid cell locations within the region of Africa where annual rainfall was >500 mm for all months from January 1983 through December 2014. Lines at top and right depict the marginal intensity by month to indicate where (latitudinally and longitudinally) intensity is most dynamic. Dotted horizontal line marks the equator.

**Figure 4:**
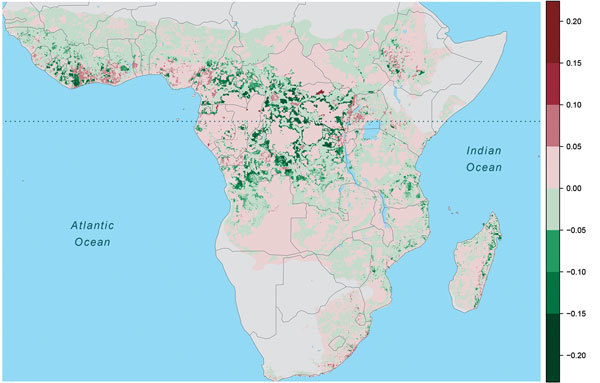
Change in annual Ebola spillover intensity (defined as average density or expected number of points per unit area and time), Africa, 1975–2015. Warm colors indicate increased spillover intensity; cool colors indicate decreased spillover intensity. Dotted horizontal line marks the equator.

**Figure 5:**
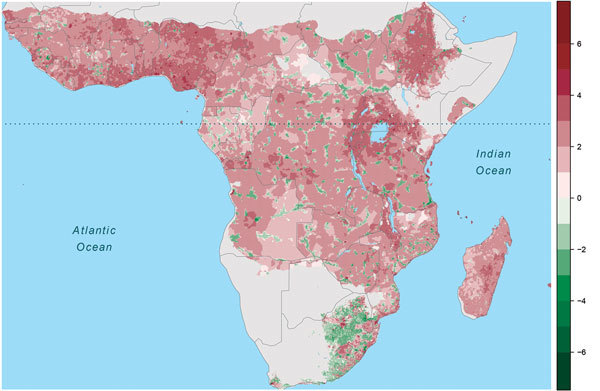
Change in human population size (log_10_/25 km^2^ grid cell), Africa, 1975–2015. Warm colors indicate increased population size, cool colors population declines. Dotted horizontal line marks the equator.

The effect of human population on Ebola spillover intensity is much smaller than climatic or seasonal effects. The change in average annual spillover intensity did not change markedly for much of Africa as population increased during 1975–2015 (Figure 4), whereas spillover intensity exhibited striking shifts with climate and seasonality (Video). Nevertheless, our model does show that spillover intensity differs by human population density. Mean annual spillover intensity was lowest where population size per 25 km^2^ grid cell was intermediate (10^2^<*x*<10^3^) and highest where population density was low (*x*<100) (Technical Appendix 2 Figure 1). Large changes in spillover intensity (±5%) during 1975–2015 appear to result mainly from population increases. In comparing 2015 to 1975 population density, shifts from intermediate-to-high population densities have generated increased Ebola spillover intensity, particularly in West Africa and the region surrounding Lake Victoria, and shifts from low-to-medium population densities have reduced spillover risk. Similarly, settlements along transportation corridors have increased in population to intermediate densities, leading to substantial declines in predicted spillover intensity. However, as a result of population consolidation over large areas of central Africa, some remote districts have declined in population, typically increasing predicted spillover intensity (Figure 4; Figure 5).

These results quantify a spatiotemporal pattern in the risk for Ebola spillover in 2 specific ways: first, as raw estimates by the model algorithm that can be directly compared between months and across locations (Figure 3); and second, as percentile ranks of these estimates or relative spillover intensity (online video). Percentile ranking adjusts for model miscalibration because some spillovers may not have been observed and because of the overrepresentation of spillover events in base logistic regression models. As such, percentile ranking preserves discriminability (i.e., classification accuracy as measured by area under the receiver-operator curve performance), even when probabilities are not well calibrated. Whether raw or ranked, spillover intensities are a measurement of risk, with values proportional to the probability of a spillover that changes as a function of environmental conditions based on the best information available on the location and timing of unique spillover events. Although the transmission, dynamics, and possibly the seasonality of different viral strains may differ, our approach, constrained by the small number of spillovers, properly considers EVD as a syndrome caused by all known strains of the Ebola virus. By constructing models to compare the covariates associated with this set of known spillover events to the background possibilities from which they might have been drawn, which we accurately approximate by using a sample of 100,000 random points, we have robustly determined how the intensity of Ebola spillovers changes with observable covariates.

## Discussion

These results indicate that 1) there is a geographic gradient of annual Ebola spillover intensity that peaks in central Africa but extends during at least some months of the year through a large portion of tropical Africa not previously considered to be at high risk (*4*,*24*), including the tropical/subtropical forest/woodland regions of Ethiopia, Angola, Zambia, East Africa, and Madagascar; 2) there is substantial seasonal fluctuation in the spatial pattern of Ebola spillover intensity; 3) there is a temporal gradient in spillover intensity in which the driest months show the lowest intensity and intensity peaks or plateaus in months of intermediate rainfall; and 4) increases in human population density may increase Ebola spillover risk in West and central Africa. Ebola spillover intensity is greatest when regions that are typically very wet make the transition to or from dry periods. This result corroborates the finding from previous studies (*7*,*11*) linking EVD events to preceding dry-to-wet transitions through time series analysis of data from a normalized difference vegetation index. Within predominantly or seasonally wet climate zones in particular, our results show Ebola spillover intensity to be highest in moderately dry months and lowest in extremely dry months.

Seasonal dynamics in spillover intensity are most pronounced where rainfall seasonality is greatest (i.e., outside the less seasonal and wetter rainforest biome of central Africa, where EVD events have been most frequent and spillover intensity is most steady throughout the year). Strong seasonal patterns may be related not only to seasonal drivers, such as rainfall, but to migration patterns and seasonal competence of wildlife reservoirs. Seasonal effects on resource availability may drive migrations or other changes in movement patterns that, in turn, may affect population density, social behaviors, and contact rates among hosts (*25*). Seasonal changes may also alter the frequency of host encounters with infective agents or material in the environment, and host immune defenses can shift with annual reproductive cycles (*26*). Seasonality is also likely to alter human behavior, including hunting effort, level of bushmeat consumption, or, more generally, the degree and kind of contact with wildlife.

Our model finds that Ebola spillover intensity varies temporally as a function of climate variables without explicitly incorporating sociocultural dimensions, such as land use, which was not available as a time series, or biotic features, such as the ranges of suspected reservoir hosts. Therefore, the degree of human disease intensity at locations far from documented EVD events may also depend on whether the range of a necessary reservoir also extends to these points. In recent work, species distribution models were used to predict the ranges of potential mammal reservoirs and the degree of overlap of predicted ranges with Ebola and Marburg spillovers to suggest likely mammalian reservoirs (*27*). Among the taxa that overlapped with all EVD sites were the sun squirrel genus (*Heliosciurus*) and the straw-colored fruit bat (*E. helvum*), both of which had predicted ranges covering nearly all of tropical Africa (with the exception of Madagascar, where *E. dupreanum* is present), where our models predicted high Ebola spillover intensity at least seasonally. Thus, our predictions across continental Africa may adequately reflect the biotic component of risk. However, an important next step would be to assess whether the presence of suitable animal hosts or cultural or socioeconomic factors in Madagascar and East Africa make this region a priority for surveillance.

Our model was trained by using great ape and human EVD events. Great ape spillover events (usually observations made by primatologists and wildlife researchers within reserves, in this dataset restricted to Gabon and the Democratic Republic of the Congo) are associated with low human population density, whereas our model associates human spillovers with high human population densities (>10^3^ persons/25 km^2^). At low population densities, epidemic spread is less likely, and deaths in remote outposts may go unreported. The link between human population density and Ebola spillover intensity could be simply a function of increased reporting at high population densities (and some locations with very low population densities). Alternatively, increased contact with or consumption of wildlife as population density increases, or perhaps the increased abundance of reservoir or bridge reservoir species at either high or low human population density (or both) could drive the relationship. Substantially increased raw Ebola spillover intensity (>5%) as a result of population increases is most apparent in areas of West Africa but could eventually include central Africa if urbanization or population consolidation continues there. We note that human population was a much less important predictor than variables capturing climate and seasonality.

In conclusion, we developed a model that predicts a pattern of widespread but seasonally very dynamic Ebola spillover intensity in savannah and humid tropical regions of Africa from the set of known spatiotemporal EVD points (n = 37 since 1990) and spatially and temporally high-resolution rainfall and population data for Africa. Ebola virus, though not the strain that led to the recent outbreak, was known to be circulating in West Africa before 2014 (*28*–*30*). However, the potential for a major human outbreak, by far the most deadly Ebola outbreak to date, was not foreseen. Answering the need for improved forecasting, surveillance, and preparation for rapid response, our model uses the best available spatiotemporal predictors and an ensemble modeling approach to accurately identify geographic regions and seasons of elevated Ebola spillover intensity, and suggests that the socio-ecologic conditions that triggered the initial spillover in Guinea may prevail over a much larger area and at a higher temporal frequency. A key public health policy implication is that some level of Ebola surveillance should be extended to regions outside of central and West Africa. Furthermore, the spatiotemporal pattern of Ebola spillover intensity we report could be used as an early warning system to inform the design of surveillance activities.

Technical Appendix 1 Dates, locations, and sources for the unique set of spillover events used in analyses of spatiotemporal fluctuations and triggers of Ebola virus disease spillover, Africa, 1961–2014.

Technical Appendix 2 Detailed methods for the determination of unique Ebola spillover events, selection of data sources and processing of geographic layers, and statistical modeling used in analyses of spatiotemporal fluctuations and triggers of Ebola virus disease spillover, Africa, 1983–2014.

Technical Appendix 3 R code used in analyses of spatiotemporal fluctuations and triggers of Ebola virus disease spillover, Africa, 1983–2014.
