# Supplementary material for: Spatiotemporal Fluctuations and Triggers of Ebola Virus Spillover
Source: Emerg Infect Dis. 2017 Mar;23(3):415–22. doi: 10.3201/eid2303.160101 (PMC5382727; doi:10.3201/eid2303.160101)
Supplement: Technical Appendix 2 — Detailed methods for the determination of unique Ebola spillover events, selection of data sources and processing of geographic layers, and statistical modeling used in analyses of spatiotemporal fluctuations and triggers of Ebola virus disease spillover, Africa, 1983–2014. [file 16-0101-Techapp-s2.pdf]

Article DOI: http://dx.doi.org/10.3201/eid2303.160101

Spatiotemporal Fluctuations and Triggers of Ebola Virus Spillover

Technical Appendix 2

Methods

Ebola Spillover Origin Points and Dates

We compiled a spatiotemporally indexed table of all known EVD events from existing sources and filtered the entries to isolate primary dates and locations of distinct spillover events (Technical Appendix 2 Figure 1). For human spillovers, we began with chronological lists compiled by the World Health Organization and U.S. Centers for Disease Control and Prevention. Key sources were Lahm et al. (2007) (*1*) and Leroy et al. (2004) (*2*) who compiled reports of wildlife mortality in Gabon and the Democratic Republic of Congo, reports by ethnologists observing great ape populations in other regions, coordinates of locations from Mylne et al. (2014) (*3*) and Kuhn's compendium (*4*).

To divide incident reports into discrete spillover events, we separated incidents into primary spillovers and secondary occurrences on the basis of widely accepted chronological, geographic or genetic distances. For example, where sequence data indicated that multiple spillover events had occurred, we considered them as such even if they overlapped spatially or temporally. Most events were reported as points. When reported as polygons we used polygon centroids as point locations. In contrast to Pigott et al. (2014) (*5*), we excluded data from sampling of healthy bats not associated with a spillover event. Critically, because we were seeking to identify potential climatological triggers, the timing of the spillover was taken to be the earliest report (often unconfirmed) of human or animal disease rather than the first date of confirmed infection in either humans or animals. Following this procedure, a primary list of 66 spatiotemporal candidate spillover points was reduced to a final list of 44 spillover events (online Technical Appendix 1, https://wwwnc.cdc.gov/EID/article/23/3/16-0101-Techapp1.xlsx).

Spatial Covariates

To exclude arid and semi-arid regions which are unlikely to harbor potential Ebola reservoir species and differ sharply in climate from locations where EVD has occurred, we defined the region of interest as the portion of Africa receiving >500 mm rainfall annually. For this region we assembled spatial data that capture the significant sources of variation in climate and land cover. Following Pigott et al. (2014) (*5*), enhanced vegetation index (EVI) and potential evapotranspiration (PET) were chosen to represent composite axes of coarse environmental variation. EVI, an optimized index derived from satellite data for vegetation monitoring, is an enhanced measurement of reflected light in the visible and near-infrared spectrum obtained by removing spectral noise caused by canopy effects and atmospheric influences. EVI is computed as:

$$EVI = G \cdot (NIR - red)/(NIR + C1 \cdot red - C2 \cdot blue + L)$$

where NIR (near-infrared), red, and blue are atmospherically-corrected for Rayleigh and ozone absorption; surface reflectance, L is the canopy background adjustment that addresses nonlinear, differential NIR and red radiant transfer through a canopy; and C1 and C2 are coefficients of aerosol resistance, using the blue band to correct for aerosol influences in the red band. The coefficients adopted in the MODIS-EVI algorithm are: L = 1, C1 = 6, C2 = 7.5, and G (gain factor) = 2.5 (*6*). EVI has been adopted by NASA as a standard product of the Moderate Resolution Imaging Spectroradiometer (MODIS) sensors. EVI values in our analyses are drawn from a raster of mean values for all months over the period 2002–2014. Mean values for EVI are served by the U.S. Geological Survey MODIS Land Processes Distributed Active Archive Center (LandDAAC) via the IRI/LDEO Climate Data Library at Columbia University (http://iridl.ldeo.columbia.edu/SOURCES/.USGS/.LandDAAC/.MODIS/.version_005/.EAF/.EVI/[X+Y+T+]average). The original spatial resolution of MODIS EVI data are 250 m, but mean values for EVI are available at 4 km resolution.

Annual PET was obtained as 30 arc-second geospatial rasters from the CGIAR Consortium for Spatial Information (http://www.cgiar-csi.org/data/global-aridity-and-pet-database) and is a measure of the ability of the atmosphere to remove water through evapo-transpiration, which is strongly correlated with climate and vegetation type. PET data were modeled using the WorldClim Global Climate Data (*6*) as input parameters. The WorldClim

data, based on a high number of climate observations and SRTM topographical data, is a high-resolution global geo-database of monthly average data (1950–2000) for precipitation, and mean, minimum and maximum temperature. PET was calculated as

PET = 0.0023 · R · (Tmean + 17.8) · Trange0.5 (mm / day),

where Tmean is mean monthly temperature, Trange is mean monthly temperature range, and R is mean monthly extra-terrestrial radiation, and validated by comparison to data from climate stations in Africa and South America. For use in statistical models to predict EVD spillover intensity, EVI and PET rasters were rescaled by subtracting the mean and dividing by the standard deviation within the rainfall >500 mm masked region.

Candidate Triggers

To characterize spatiotemporal variation at seasonal, inter-annual, and decadal scales, we compiled the following datasets.

1) Population count grids for Africa for 1960, 1970, 1980, 2000, 2005, 2010, and 2015 at 2.5 arc-minutes scale (~25 km^2 at the equator) from the Gridded Population of the World version 3 (*7*), produced by the Columbia University Center for International Earth Science Information Network (CIESIN) and available from the Socioeconomic Data and Applications Center (SEDAC), one of the Distributed Active Archive Centers (DAACs) in the Earth Observing System Data and Information System (EOSDIS) of the U.S. National Aeronautics and Space Administration (NASA). Data for each decade 1960–2000 reflect national or subnational input administrative units of varying resolution depending on original reporting, whereas 2005, 2010, and 2015 grids were extrapolated by CIESIN based on a combination of sub-national growth rates from census dates and national growth rates from United Nations statistics. We linearly interpolated counts by cell for intervening years. Guided by exploratory analyses of the data using boosted regression trees that identified a nonlinear relationships between Ebola spillover intensity and population size, we log10-transformed values of human population and grouped into three bins according to $x \leq 10^2$, $10^2 < x < 10^3$, $x \geq 10^3$.

2) Monthly rainfall was aggregated from daily rainfall estimates obtained from the Rainfall Estimator (RFE) (*8*), a data product developed in 1998 by the Climate Prediction Center (CPC) at the National Oceanic and Atmospheric Administration (NOAA) to support accurate monitoring of large-scale and climatic trends with a high (0.1°) spatial resolution by blending

gauge and satellite information on a near-real time basis. This dataset provides daily rainfall estimates over the African continent for the time period January 1983-present (http://www.cgiar-csi.org/data/global-aridity-and-pet-database).

3) In addition to actual monthly rainfall, as a means of incorporating the potential importance of relative rainfall, we created a rainfall anomaly index as follows. For the series of 384 monthly rainfall rasters, we divided the value of each month-location by the maximum value for that location to create a set of 384, scaled rasters corresponding to the original monthly rainfall rasters.

Model Fitting and Validation

Analysis was restricted to the 37 out of 44 (80.5%) EVD events occurring since 1982, the period for which monthly rainfall estimates for Africa were available. These were divided into 2/3 for training and 1/3 for testing. Because actual monthly rainfall at month and site of EVD spillovers varied considerably, we stratified by rainfall, first ranking points by rainfall amount and then assigning every third point to the test set. A set of 100,000 random background points was sampled from the 1,115,874 × 384 location-time combinations within the >500 mm rainfall mask (30 arc second resolution). Background points were similarly divided into 2/3 for training and 1/3 for testing. For spillover and background points, values from each covariate raster extracted to presence and background points. To test the sensitivity of model results to the choice of background points, we ran the models again on a second set of random points. For each set of background points, receiver operator characteristic (ROC) curves (Technical Appendix 2 Figure 2) visualize classification error for 1) prediction on training and test data for models derived from analyses using all spillover points, and 2) prediction on training data for models derived from analyses using human spillovers only. Area under the ROC curve, a metric of model performance, was similar for the models using human spillover points only (0.84 versus 0.83 in one iteration, 0.83 in both cases for the second set of random background points) when predicted on training data. Too few human spillover points were available to hold out a subset for testing. Overall, comparison of model results from two separate sets of random background points indicates little sensitivity to choice of background points (Technical Appendix 2 Figure 2), suggesting that the range of spatiotemporal variability is adequately captured by randomly sampling 100,000 background points over the 384 months for the masked region of Africa.

Model Performance Testing

Exploratory analysis showed that conventional, flexible machine learning algorithms, such as boosted regression trees had a strong tendency to overfit to the data, very likely because of the very small number of points available for fitting. Because “bagged” low-variance models have previously been shown to yield reasonable generalizability (*9*), we reasoned that bagged ensembles of more rigid models might yield superior predictive performance. Bagging (bootstrap aggregating) is a machine learning approach that makes use of the predictive power generated from ensembles of so-called “weak” learners, i.e., weakly tuned models based on small subsets of the data (*10*). For this reason, we modeled EVD spillover intensity using bagged logistic regression models with main effects only. Using all 5 predictors, we fit 1000 models in which we randomly sampled 10 of the 22 outbreaks in the training dataset and 100 of 100,000 training background points. We predicted each of the 1000 fitted models on both the training and test data. We compared mean predicted values for training and test points to EVD versus background labels in each dataset to calculate AUC, the area under the receiver operating characteristic curve (ROC). ROC curves plot the performance of a binary classifier system as its discrimination threshold varies. By plotting the true positive rate as a function of the false positive rate, AUC provides an index of classification accuracy such that an AUC score of 0.5 indicates a classifier that performs no better than random, and an AUC score of 1 indicates perfect discrimination.

Risk Mapping

Following the bagging procedure above, we used the complete dataset (37 spillover, 100,000 background points) to predict EVD spillover intensity across the entire masked (>500 mm rainfall) region of Africa for all 384 months (January, 1983-present for which gridded rainfall data was available from NOAA) using human population estimates for 2015. We then averaged the resulting 384 spillover intensity rasters by month to create a mapped visualization of seasonal shifts in spillover intensity across Africa. This procedure was repeated using human population estimates for 1975. To map changes in EVD spillover intensity as a function of changes in human population across 4 decades, we averaged predicted spillover intensity across all months for 1975 and 2015 and took the difference of the 2 resulting grids.

References

1. Lahm SA, Kombila M, Swanepoel R, Barnes RFW. Morbidity and mortality of wild animals in relation to outbreaks of Ebola haemorrhagic fever in Gabon, 1994-2003. Trans R Soc Trop Med Hyg. 2007;101:64–78. PubMed http://dx.doi.org/10.1016/j.trstmh.2006.07.002

2. Leroy EM, Telfer P, Kumulungui B, Yaba P, Rouquet P, Roques P, et al. A serological survey of Ebola virus infection in central African nonhuman primates. J Infect Dis. 2004;190:1895–9. PubMed http://dx.doi.org/10.1086/425421

3. Mylne A, Brady OJ, Huang Z, Pigott DM, Golding N, Kraemer MU, et al. A comprehensive database of the geographic spread of past human Ebola outbreaks. Sci Data. 2014;1:140042. PubMed http://dx.doi.org/10.1038/sdata.2014.42

4. Kuhn JH. Filoviruses: a compendium of 40 years of epidemiological, clinical, and laboratory studies. New York: Springer; 2008.

5. Pigott DM, Golding N, Mylne A, Huang Z, Henry AJ, Weiss DJ, et al. Mapping the zoonotic niche of Ebola virus disease in Africa. eLife. 2014;3:e04395. PubMed http://dx.doi.org/10.7554/eLife.04395

6. Hijmans RJ, Cameron SE, Parra JL, Jones PG, Jarvis A. Very high resolution interpolated climate surfaces for global land areas. Int J Climatol. 2005;25:1965–78. http://dx.doi.org/10.1002/joc.1276

7. Center for International Earth Science Information Network. Gridded Population of the World, Version 3 (GPWv3): Population Count Grid. Palisades, NY: NASA Socioeconomic Data and Applications Center [cited 2014 Sep 1]. http://dx.doi.org/10.7927/H4639MPP

8. Herman A, Kumar VB, Arkin PA, Kousky JV. Objectively determined 10-day African rainfall estimates created for famine early warning systems. Int J Remote Sens. 1997;18:2147–59. http://dx.doi.org/10.1080/014311697217800

9. Valentini G, Dietterich TG. Low bias bagged support vector machines. In: Proceedings of the 20th International Conference on Machine Learning (ICML-2003), Washington DC; 2003. p. 752–9 [cited 2014 Dec 15]. http://www.aaai.org/Papers/ICML/2003/ICML03-098.pdf

10. Breiman L. Bagging predictors. Mach Learn. 1996;24:123–40. http://dx.doi.org/10.1007/BF00058655

11. Huete A, Didan K, Miura T, Rodriguez EP, Gao X, Ferreira LG. Overview of the radiometric and biophysical performance of the MODros Inf Serv. vegetation indices. Remote Sens Environ. 2002;83:195–213. http://dx.doi.org/10.1016/S0034-4257(02)00096-2

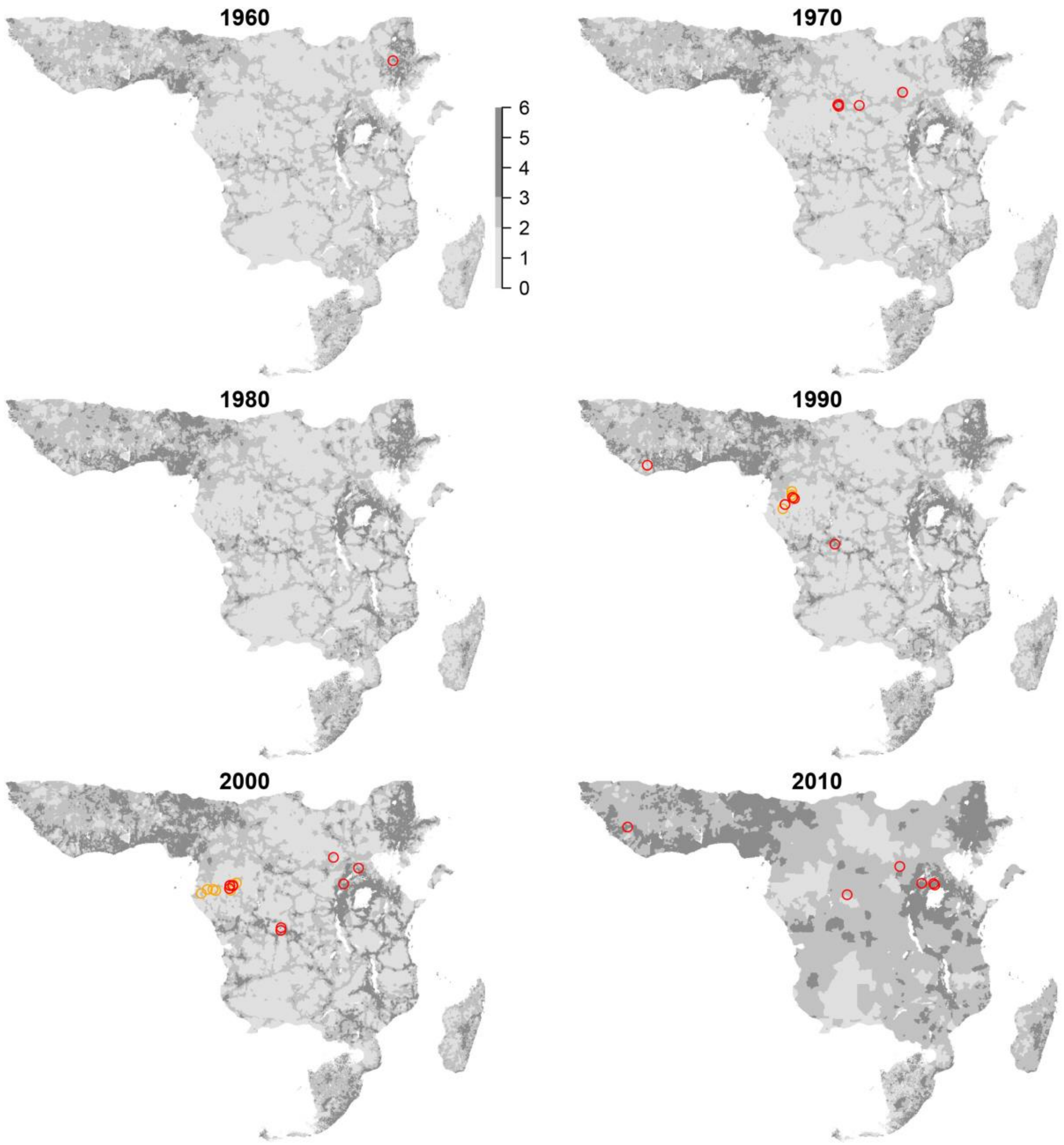

Technical Appendix 2 Figure 1. Population density and Ebola spillover locations for the masked region of Africa (>600 mm annual rainfall) for each decade during 1960–2010. Legend numbers represent human population ranges as powers of 10 per 25 km^2. Red circles mark human spillovers, orange circles mark nonhuman primate and other mammal spillovers.

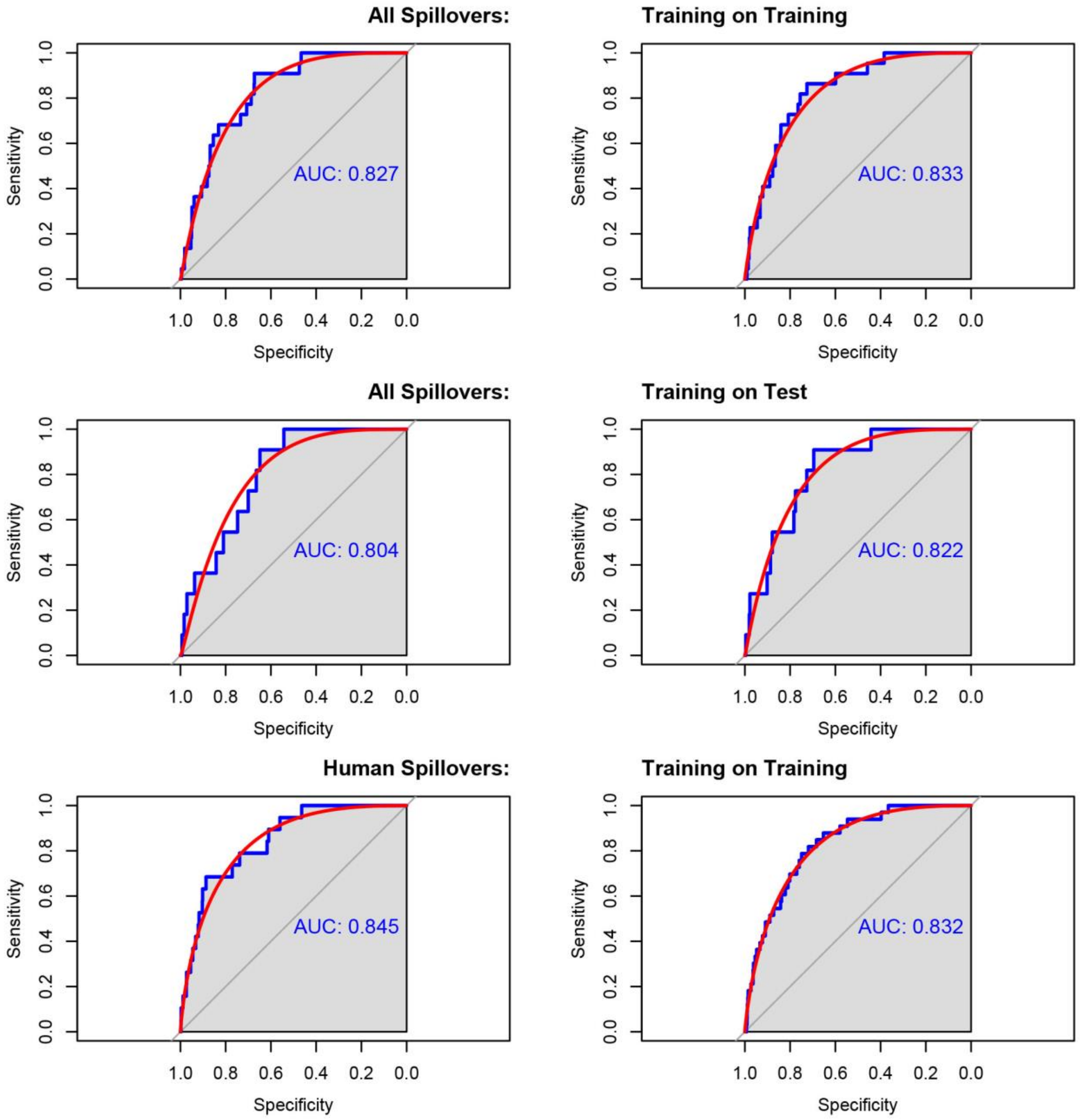

Technical Appendix 2 Figure 2. Receiver operator characteristic curves showing performance of models resulting from analyses using all Ebola virus disease spillover points, predicted on 2/3 training data (top), and predicted on holdout 1/3 test data (middle), Africa, 1983–2015. Bottom row shows performance of models resulting from analyses using human spillover points only predicted on training data. Left and right columns compare results from 2 different sets of background points. Filled polygons represent the area under the receiver operator characteristic curve; red arcs are smoothed curves.
