# Supplementary material for: Spatiotemporal Fluctuations and Triggers of Ebola Virus Spillover
Source: Emerg Infect Dis. 2017 Mar;23(3):415–22. doi: 10.3201/eid2303.160101 (PMC5382727; doi:10.3201/eid2303.160101)
Supplement: Technical Appendix 3 — R code used in analyses of spatiotemporal fluctuations and triggers of Ebola virus disease spillover, Africa, 1983–2014. [file 16-0101-Techapp-s3.pdf]

Article DOI: http://dx.doi.org/10.3201/eid2303.160101

Spatiotemporal Fluctuations and Triggers of Ebola Virus Spillover

Technical Appendix 3

R Code Used for Risk Mapping

```
---
title: "ebola.final.spillover.Rmd"
output: word_document
---
First, we load index points of unique Ebola spillover events as determined by Laura Alexander.
```{r}
# index.pts <- as.data.frame(read.table("index.pts.csv",header = T, sep=",",na.strings=c("na","NA")))
```
Subset Ebola spillovers occurring since 1983, and that were the earliest reported location for each spillover event.
```{r}
# index.pts2 <- index.pts[ which(index.pts$year > 1983 & index.pts$initial == "TRUE"), ]
```
Load R packages needed for all the data development and analysis. (If these packages are not currently installed,
they will need to be.)
```{r}
library(raster)
library(sp)
library(maptools)
library(rgdal)
library(gdalUtils)
library(dismo)
library(rworldmap)
library(grid)
library(latticeExtra)
```
Get lat-long extent for Africa.
```{r}
# africa.extent<-extent(c(-17.30253,55.84747,-34.83332,16.05834))
```
Load rainfall in mm from WorldClim 1.4 from http://www.worldclim.org/current. Values are averages over 1960-1990 for
each month.
```{r}
# jan.rf<-raster("prec_1.bil")
# feb.rf<-raster("prec_2.bil")
# mar.rf<-raster("prec_3.bil")
# apr.rf<-raster("prec_4.bil")
# may.rf<-raster("prec_5.bil")
# jun.rf<-raster("prec_6.bil")
# jul.rf<-raster("prec_7.bil")
# aug.rf<-raster("prec_8.bil")
# sep.rf<-raster("prec_9.bil")
# oct.rf<-raster("prec_10.bil")
# nov.rf<-raster("prec_11.bil")
# dec.rf<-raster("prec_12.bil")
```
Crop monthly rainfall rasters to Africa.
```{r}
# af.rf.jan<-crop(jan.rf,africa.extent)
# af.rf.feb<-crop(feb.rf,africa.extent)
# af.rf.mar<-crop(mar.rf,africa.extent)
# af.rf.apr<-crop(apr.rf,africa.extent)
# af.rf.may<-crop(may.rf,africa.extent)
# af.rf.jun<-crop(jun.rf,africa.extent)
# af.rf.jul<-crop(jul.rf,africa.extent)
# af.rf.aug<-crop(aug.rf,africa.extent)
# af.rf.sep<-crop(sep.rf,africa.extent)
# af.rf.oct<-crop(oct.rf,africa.extent)
# af.rf.nov<-crop(nov.rf,africa.extent)
# af.rf.dec<-crop(dec.rf,africa.extent)
```
Create stack of average rainfall over Africa by month.
```

```{r}
# af.rf.stack<-
stack(af.rf.jan,af.rf.feb,af.rf.mar,af.rf.apr,af.rf.may,af.rf.jun,af.rf.jul,af.rf.aug,af.rf.sep,af.rf.oct,af.rf.nov,af.
rf.dec)
# af.rf.brick<-brick(af.rf.stack)
# writeRaster(af.rf.brick,filename="af.rf.brick.grd",overwrite=TRUE)
# af.rf.brick<-brick("af.rf.brick.grd")
```
Sum stack to get average annual rainfall for Africa.
```{r}
# af.rf.annual<-sum(af.rf.stack)
```
Create mask region where annual rainfall >500 mm in Africa.
```{r}
# m<-c(0,500,NA, 500,4559,1)
# rf.mask.500<-reclassify(af.rf.annual,m)
# writeRaster(rf.mask.500,"rf.mask.500.tif")
# rf.mask.500<-raster("rf.mask.500.tif")
```
Set random seed and generate random background points from within the rainfall mask. To test the effect of the choice of background points, we also generated a second set of background point using set.seed(45).
Set random seed.
```{r}
# randomseed<-seq.int(1,528,1)
# set.seed(55)
```
Get 100k random background points from with the >500 mm rainfall mask for Africa.
```{r}
# bg<-randomPoints(mask, 100000)
```
Set id for each point.
```{r}
# id<-seq.int(44,100000,1)
```
Join id to coordinates of the point.
```{r}
# bg<-cbind(bg,id)
```
Create sequence of years from 1983 through 2014, and months 1-12.
```{r}
# years<-seq.int(1983,2014,1)
# months<-seq.int(1,12,1)
```
Generate a random year and month (1970-2014 or 1983-2014) for each background point.
```{r}
# year<-NULL
# month<-NULL
# for (i in 1:100000) {
# y<-sample(years,1)
# m<-sample(months,1)
# year<-rbind(year,y)
# month<-rbind(month,m)
# }
```
Set presence = 0 for all background points.
```{r}
# pres<-rep(0,100000)
# all.bg<-cbind(bg,year,month,pres)
```
Set column headings for coordinates, id, year, month and presence.
```{r}
# colnames(all.bg)<-c("x","y","id","year","month","pres")
```
Combine spillover locations with background points.
```{r}
# all.pts<-rbind(index.pts2[,1:6],all.bg)
```
Take daily data from the Rainfall Estimator and aggregates it to monthly to create a stack of rainfall grids by month for the period 1983-2014.
Source: "In 1998, the Climate Prediction Center (CPC) developed the Rainfall Estimator (RFE) (Herman et. al. 1997) in response to the need for higher resolution operational daily rainfall estimates to support the humanitarian aid programs of USAID / Famine Early Warning Systems Network (FEWS - NET). The RFE hascontinued to provide an accurate monitoring of large-scale and regional climatic and hydrological trends. It is a unique product compared to other satellite rainfall estimators because of its high, 0.1° gridded spatial resolution,and its ability to blend gauge and satellite information on a near-real time basis to provide daily (06Z-06Z) rainfall estimates over the African continent" (http://www.cpc.ncep.noaa.gov/products/fews/AFR_CLIM/AMS_ARC2a.pdf). The daily data above was aggregated to derive monthly rainfall estimates at the same spatial resolution. A number of days in the early period 1983-1985 were missing data for many days.
Loop through all of the daily rasters to create a raster aggregating daily values to monthly values.
```{r}
# year<-
c("1983","1984","1985","1986","1987","1988","1989","1990","1991","1992","1993","1994","1995","1996","1997","1998","1999
","2000","2001","2002","# month<-c("01","02","03","04","05","06","07","08","09","10","11")
# day<-
c("01","02","03","04","05","06","07","08","09","10","11","12","13","14","15","16","17","18","19","20","21","22","23","2
4","25","26","27","# monthsum<-stack()
# for (y in 1:10){
# for (m in 1:12){
```

````
# for (d in 1:31){
# if (exists(paste("africa_arc.", year[y], month[m],day[d], ".tif", sep="")))
# dia<-raster(paste("africa_arc.", year[y], month[m],day[d], ".tif", sep=""))
# monthsum<-sum(stack(monthsum,dia))
# }
# writeRaster(monthsum, paste("/Google Drive/ebola/monthyear/africa_prcp", year[y], month[m],
".tif",sep=""),overwrite=TRUE)
# }
# }
```
Create a raster stack of rainfall grids by month for the period 1983-2014.
```{r}
# setwd("~/Google Drive/ebola/monthyear")
# f <- list.files()
# r <- raster(f[1])
# rf.stack <- stack(r)
```
Bring in population count grids for Africa for 1960, 1970, 1980, 2000, 2005, 2010, and 2015 (the last 3 estimated)
from http://sedac.ciesin.columbia.edu/data/set/grump-v1-population-density/maps?facets=region:africa.
```{r}
# pop60<-raster("afpop60.tif")
# crs(pop60)<-"+proj=longlat +datum=WGS84 +no_defs +ellps=WGS84 +towgs84=0,0,0"
# pop70<-raster("afpop70.tif")
# crs(pop70)<-"+proj=longlat +datum=WGS84 +no_defs +ellps=WGS84 +towgs84=0,0,0"
# pop80<-raster("afpop80.tif")
# crs(pop80)<-"+proj=longlat +datum=WGS84 +no_defs +ellps=WGS84 +towgs84=0,0,0"
# pop90<-raster("afpop90.tif")
# crs(pop90)<-"+proj=longlat +datum=WGS84 +no_defs +ellps=WGS84 +towgs84=0,0,0"
# pop00<-raster("afpop00.tif")
# crs(pop00)<-"+proj=longlat +datum=WGS84 +no_defs +ellps=WGS84 +towgs84=0,0,0"
# pop05<-raster("afpop05.tif")
# crs(pop05)<-"+proj=longlat +datum=WGS84 +no_defs +ellps=WGS84 +towgs84=0,0,0"
# pop10<-raster("afpop10.tif")
# crs(pop10)<-"+proj=longlat +datum=WGS84 +no_defs +ellps=WGS84 +towgs84=0,0,0"
# pop15<-raster("afpop15.tif")
# crs(pop15)<-"+proj=longlat +datum=WGS84 +no_defs +ellps=WGS84 +towgs84=0,0,0"
```
Mask out no data areas which are calced to 0 in the orginial files so that 0 and no data are distinct values.
```{r}
# pop60<-mask(pop60,mask)
# pop70<-mask(pop70,mask)
# pop80<-mask(pop80,mask)
# pop90<-mask(pop90,mask)
# pop00<-mask(pop00,mask)
# pop05<-mask(pop05,mask)
# pop10<-mask(pop10,mask)
# pop15<-mask(pop15,mask)
```
Crop grids to the extent of the >600 mm rainfall mask.
```{r}
# pop60<-crop(pop60,mask)
# pop70<-crop(pop70,mask)
# pop80<-crop(pop80,mask)
# pop90<-crop(pop90,mask)
# pop00<-crop(pop00,mask)
# pop05<-crop(pop05,mask)
# pop10<-crop(pop10,mask)
# pop15<-crop(pop15,mask)
```
Change 0 values for population to 1 to avoid generating NAs with log transformation.
```{r}
# pop60<-calc(pop60,fun=function(x){ x[x==0] <- 1; return(x)})
# pop70<-calc(pop70,fun=function(x){ x[x==0] <- 1; return(x)})
# pop80<-calc(pop80,fun=function(x){ x[x==0] <- 1; return(x)})
# pop90<-calc(pop90,fun=function(x){ x[x==0] <- 1; return(x)})
# pop00<-calc(pop00,fun=function(x){ x[x==0] <- 1; return(x)})
# pop05<-calc(pop05,fun=function(x){ x[x==0] <- 1; return(x)})
# pop10<-calc(pop10,fun=function(x){ x[x==0] <- 1; return(x)})
# pop15<-calc(pop15,fun=function(x){ x[x==0] <- 1; return(x)})
```
Log 10 transform population values of population rasters.
```{r}
# logpop60<-calc(pop60, fun=function(x){log10(x)})
# logpop70<-calc(pop70, fun=function(x){log10(x)})
# logpop80<-calc(pop80, fun=function(x){log10(x)})
# logpop90<-calc(pop90, fun=function(x){log10(x)})
# logpop00<-calc(pop00, fun=function(x){log10(x)})
# logpop05<-calc(pop05, fun=function(x){log10(x)})
# logpop10<-calc(pop10, fun=function(x){log10(x)})
# logpop15<-calc(pop15, fun=function(x){log10(x)})
```
Create population density stack.
```{r}
# popstack<-stack(logpop60,logpop70,logpop80,logpop90,logpop00,logpop05,logpop10,logpop15)
# crs(popstack)<-"+proj=longlat +datum=WGS84 +no_defs +ellps=WGS84 +towgs84=0,0,0"
```
Create population density dataframe from the raster stack.
```{r}
````

`````
# pop.df<-as.data.frame(popstack,xy=TRUE)
```
Name columns by year.
```{r}
# colnames(pop.df)<-c("x","y",1960,1970,1980,1990,2000,2005,2010,2015)
```
For all years between censuses, linearly interpolate values for population density for each cell.
Get the difference in years between each population estimate
```{r}
# pop.estimates <- c(1960,1970,1980,1990,2000,2005,2010,2015)
# d <- diff(pop.estimates)
```
Get the year columns from the dataframe and linearly interpolate between years.
```{r}
# pop.df2<-pop.df[,3:10]
# tvseq <- function(...)t(Vectorize(seq.default)(...))
# L <- lapply(seq(d), function(i) tvseq(from=pop.df2[,i], to=pop.df2[,i+1], length.out=d[i]+1)[,-1])
```
Combine columns for interpolated years to census years.
```{r}
# pop.df3 <- cbind(pop.df2[,1],do.call(cbind, L))
```
Leave out 2015 and add coordinates to dataframe.
```{r}
# pop.df4<-cbind(pop.df2[,1:2],pop.df3[,1:55])
```
Convert population dataframe back to a raster stack by year for each cell.
Turn dataframe into a gridded dataframe.
```{r}
# coordinates(pop.df4) <- ~ x + y
# gridded(pop.df4) <- TRUE
```
Create a raster stack of log10-transformed population counts for every year from 1983 through 2014, the years for
which gridded monthly rainfall are available.
```{r}
# popstack<-raster(pop.df4[,15])
# for (i in 24:ncol(pop.df4)){
# pop<-raster(pop.df4[,i])
# popstack<-addLayer(popstack,pop)
# print(nlayers(popstack))
# }
# crs(popstack)<-"+proj=longlat +datum=WGS84 +no_defs +ellps=WGS84 +towgs84=0,0,0"
# popbrick<-brick(popstack)
# writeRaster(popbrick,filename="popbrick.grd",overwrite=TRUE)
# popbrick<-brick("popbrick.grd")
```
Extract rainfall data to presence and background points with year and month values between January 1983 to September
2014, the period for which gridded rainfall data is available. Also, derive the scaled rainfall value (relative to
the maximum at a cell) for each month-site.
```{r}
# library(matrixStats)
# years<-seq.int(1983,2014,1)
# months<-rep(seq(1,12,1),32)
# yrs<-sort(rep(years*100,12))
# m.yrs<-colSums(rbind(months,yrs))
# num<-seq(1,384,1)
# lookup<-as.data.frame(cbind(m.yrs,num))
# yr.m.table<-all.pts[,3:4]
# yr.m.table<-unique(yr.m.table)
# rf.yr.m.cols<-NULL
# for (i in 1:nrow(yr.m.table)){
# yr<-yr.m.table[i,1]
# yr.num<-(yr - 1982)
# xy.coords<-all.pts[i,1:2]
# m<-yr.m.table[i,2]
# yr.m<-as.numeric(yr*100+m)
# ind<-which(lookup[,1]==yr.m, arr.ind=TRUE)
# name <- paste("rainfall/africa_prcp", yr.m, "_Resample_R.tif",sep = "")
# r<-raster(name)
# yr.pts<-subset(all.pts,year==yr)
# yr.month.pts<-subset(yr.pts,month==m)
# # use coordinates to extract values at points
# rf.ym<-extract(r,yr.month.pts[,1:2])
# rf.ym.log<-log10(rf.ym+1)
# # now get scaled rainfall
# # extract all values for a cell
# rf.site<-extract(rf.stack,yr.month.pts[,1:2])
# rf.max<-rowMaxs(rf.site)
# rf.rescaled<-rf.ym/rf.max
# # now put it all together in a row
# rf.yr.m<-cbind(yr.month.pts,rf.ym.log,rf.rescaled)
# # now concatenate rows
# rf.yr.m.cols<-rbind(rf.yr.m.cols,rf.yr.m)
# print(i)
# }
```
Get population value at each site for the year corresponding to the point.
```{r}
`````

```
# yr.table<-unique(yr.table)
# pd.yr.row<-NULL
# rf.pd.cols<-NULL
# for (i in 1:32){
# yr<-years[i]
# yr.num<-(yr - 1982)
# yr.pts<-rf.yr.m.cols[which(rf.yr.m.cols$year==yr),]
# yr.raster<-subset(popbrick,yr.num)
# pd.yr<-extract(yr.raster,yr.pts[,1:2])
# pd.yr.row<-cbind(yr.pts,pd.yr)
# rf.pd.cols<-rbind(rf.pd.cols,pd.yr.row)
# }
```
Create 3 bins of log10 population, 0-2, 2-3, and 3-6.
```{r}
# pd.yr.binned<-as.numeric(cut(rf.pd.cols[,9], breaks = c(0,2,3,6)))
```
Function to rescale a raster by subtracting mean and dividing by standard deviation.
```{r}
# rescale.raster <- function(data0, data1=NULL){
# if(missing(data1)){
# raster <- (data0-cellStats(data0,'mean'))/cellStats(data0,'sd')
# }else{
# raster <- (data1-cellStats(data0,'mean'))/cellStats(data0,'sd')
# }
# return(raster)
# }
```
Extract values of the enhanced vegetation index (EVI) available from by the U.S. Geological Survey MODIS Land Processes Distributed Active Archive Center (LandDAAC) via the IRI/LDEO Climate Data Library at Columbia University (http://iridl.ldeo.columbia.edu/SOURCES/.USGS/.LandDAAC/.MODIS/.version_005/.EAF/.EVI/[X+Y+T+]average/).
Because EVI scales over a large range, values were rescaled by subtracting the mean and dividing by the standard deviation within the rainfall >600 mm masked region using the function above.
```{r}
# evir<-raster("evir.tif")
# evi<-extract(evir,dyn.cols[,1:2])
```
Extract values of Annual potential evapotranspiration (PET) obtained as 30 arc-second geospatial rasters from the CGIAR Consortium for Spatial Information (http://www.cgiar-csi.org/data/global-aridity-and-pet- database).
Because PET scales over a large range, values were rescaled by subtracting the mean and dividing by the standard deviation within the rainfall >600 mm masked region using the function above.
```{r}
# petr<-raster("petr.tif")
# pet<-extract(petr,dyn.cols[,1:2])
```
Put all the data together.
```{r}
# final.pts<-cbind(rf.pd.cols,pd.yr.binned,evi,pet)
# final.pts<-as.data.frame(read.table("final.pts.csv",header = T, sep=",",na.strings=c("na","NA")))
```
Subset presence points.
```{r}
pres.pts<-subset(final.pts,pres==1)
```
Sort by rainfall amount.
```{r}
o<-order(pres.pts[,7])
pres.pts<-pres.pts[o,]
```
Set rows for training and test, stratifying for rainfall amount by choosing every 3rd point for test.
```{r}
train.rows<-c(1,2,4,5,7,8,10,11,13,14,16,17,19,20,22,23,25,26,28,29,31,32)
test.rows<-c(3,6,9,12,15,18,21,24,27,30,33)
```
Create training and test presence points.
```{r}
train.pres<-pres.pts[train.rows,]
test.pres<-pres.pts[test.rows,]
```
Subset absence points.
```{r}
abs.pts<-as.data.frame(subset(final.pts,pres==0))
```
Randomly split absence points using random seed for repeatability. "index/3"" indicates randomly divide into 3/3 training and 1/3 test.
```{r}
splitdf <- function(dataframe, seed=NULL) {
if (!is.null(seed)) set.seed(seed)
index <- 1:nrow(dataframe)
trainindex <- sample(index, trunc(length(index)/3))
trainset <- dataframe[-trainindex, ]
testset <- dataframe[trainindex, ]
list(trainset=trainset,testset=testset)
}
```
Split using random seed = 59.
```{r}
abs.index<-splitdf(abs.pts,59)
```

````
```
Create training and test background sets.
```{r}
train.bg<-abs.index$trainset
test.bg<-abs.index$testset
```
New packages to do bagged logistic regression.
```{r}
library(parallel)
library(foreach)
library(doSNOW)
library(rlecuyer)
library(data.table)
```
Bagging function.
```{r}
bagging<-function(form.x.y,train.pres,train.bg,new.data,iterations=1000){
predictions<-foreach(m=1:9,.combine='+') %dopar% {
training_positions.p <- sample(nrow(training.pres),size=10)
training_positions.b <- sample(nrow(training.abs),size=100)
train_pos.p<-1:nrow(training.pres) %in% training_positions.p
train_pos.b<-1:nrow(training.abs) %in% training_positions.b
glm_fit<-glm(form.x.y,data=rbind(training.pres[train_pos.p,],training.abs[train_pos.b,]),family=binomial(logit))
predict(glm_fit,newdata=new.data,type="response")
}
return(predictions)
}
```
Allocate memory.
```{r}
cores.to.use<-176
cl<-makeCluster(cores.to.use,type="MPI")
clusterSetupRNG(cl)
registerDoSNOW(cl)
```
Logistic regression formula.
```{r}
glm.formula<-as.formula("pres~rf.ym+evi+pet+rf.scaled+as.factor(pd.yr.binned)")
```
Predict bagged model on training set.
```{r}
training<-rbind(train.pres,train.bg)
output1<-
bagging(form.x.y=glm.formula,training.pres=train.pres,training.abs=train.bg,new.data=training,iterations=cores.to.use)
```
Predict bagged model on test set.
```{r}
testing<-rbind(test.pres,test.bg)
output2<-
bagging(form.x.y=glm.formula,training.pres=train.pres,training.abs=train.bg,new.data=testing,iterations=cores.to.use)
```
Now do PREDICTION on the entire area of Africa >500 mm rainfall using bagged model and full data.
Create a lattice of points from the masked region for spillover intensity prediction. Note resolution reduced by a
factor of 4 to speed computation.
First, resample the >500 mm rainfall mask.
```{r}
resampleFactor<-4
inCols <- ncol(mask)
inRows <- nrow(mask)
resampledRaster <- raster(ncol=(inCols / resampleFactor), nrow=(inRows / resampleFactor))
extent(resampledRaster) <- extent(mask)
```
Turn cells within the >500 mm rainfall portion of the new raster into points.
```{r}
coord.raster<-subset(resampledRaster,1)
coord.db<-as.data.frame(coord.raster,xy=TRUE)
coords<-coord.db[,1:2]
```
Create a raster stack of rainfall for the >500 mm of rainfall region for the 384 months of the 1983-2014 period.
```{r}
# setwd("~/rainfall/")
# f<-list.files()
# rf.rasters <- lapply(f,raster)
```
Extract rainfall values at the resampled rainfall mask coordinates, and turn into a dataframe.
```{r}
# rf.extracted <- lapply(rf.rasters,extract,coords)
# rf.extracted2 <- unlist(rf.extracted)
# setwd("~/")
# rf.ym.df<-as.data.frame(rf.extracted2)
# rf.ym.df<-as.data.frame(read.table("rf.ym.df.csv",header = T, sep=",",na.strings=c("na","NA")))
```
Generate scaled rainfall (percent of maximum value at a cell 1983-2014) dataframe from rainfall dataframe.
```{r}
library(matrixStats)
# rf.ym.all<-as.matrix(rf.ym.df)
# rf.maxes.all<-rowMaxs(rf.ym.all,na.rm=T)
# rf.scaled.all<-rf.ym.all/rf.maxes.all
````

```
# rf.scaled.all<-as.data.frame(read.table("rf.scaled.all.csv",header = T, sep=",",na.strings=c("na","NA")))
```
Extract values for static variables, EVI and PET, and 2015 values of binned population. Combine into one dataframe.
```{r}
# evi.all<-extract(evir,coords)
# pet.all<-extract(petr,coords)
```
Extract 2015 population counts at the resampled rainfall mask coordinates.
```{r}
# pd.yr.all<-extract(logpop15,coords)
```
Bin population values.
```{r}
# pd.yr.binned.all<-as.numeric(cut(pd.yr, breaks = c(0,2,3,6)))
```
Combine EVI, PET, and binned population into a single dataframe.
```{r}
# evi.pet.pd.binned<-cbind(evi.all,pet.all,pd.yr.binned.all)
# evi.pet.pd.binned<-as.data.frame(read.table("evi.pet.pd.binned.csv",header = T, sep=",",na.strings=c("na","NA")))
```
Combine training and test data for new bagged logistic regression model to do prediction on >500 mm of rainfall masked region of Africa.
```{r}
# final.p<-rbind(train.pres,test.pres)
# final.b<-rbind(train.bg,test.bg)
```
Loop to do bagging predictions on all 384 months of rainfall. Do in 12 parts because loading data takes the same amount of time.
```{r}
# for (i in 1:12){
# rf.scaled<-fread("rf.scaled.all.csv",select=((i*32)-31):(i*32))
# rf.ym<-fread("rf.ym.df.csv",select=((i*32)-31):(i*32))
# for (j in 1:32){
# rf.scaled.step<-rf.scaled.all[,j] #Single year of data
# rf.ym.step<-rf.ym.all[,j] #Single year of data
# all.data.step<-as.data.frame(cbind(evi.pet.pd.binned,rf.scaled.step,log10(rf.ym.step+1)))
# colnames(all.data.step)<-c("evi","pet","pd.yr.binned","rf.scaled","rf.ym")
# sum<-NULL
# chunks<-6
# for (k in 1:chunks){
# output<-
bagging(form.x.y=glm.formula,train.pres=final.p,training.bg=final.b,new.data=all.data.step,iterations=cores.to.use)
# sum<-sum+output
# }
# mean.predict<-sum/(cores.to.use*chunks)
# writeRDS(mean.predict,file=paste("Bags/predictionsSet",i*j))
# print(i*j)
# }
# }
```
Create sequence from 1-384 for each month.
```{r}
# jan<-seq(1,384,12)
# feb<-seq(2,384,12)
# mar<-seq(3,384,12)
# apr<-seq(4,384,12)
# may<-seq(5,384,12)
# jun<-seq(6,384,12)
# jul<-seq(7,384,12)
# aug<-seq(8,384,12)
# sep<-seq(9,384,12)
# oct<-seq(10,384,12)
# nov<-seq(11,384,12)
# dec<-seq(12,384,12)
```
Separate Ebola spillover intensity values by month.
```{r}
library(matrixStats)
# jan.risk<-NULL
# for (i in 1:length(jan)){
# risk<-readRDS(paste("Bags/predictionsSet",jan[i],".rds",sep=""))
# jan.risk<-cbind(jan.risk,risk)
# jan.risk<-rowSums(jan.risk)
# }
# feb.risk<-NULL
# for (i in 1:length(feb)){
# risk<-readRDS(paste("Bags/predictionsSet",feb[i],".rds",sep=""))
# feb.risk<-cbind(feb.risk,risk)
# feb.risk<-rowSums(feb.risk)
# }
# mar.risk<-NULL
# for (i in 1:length(mar)){
# risk<-readRDS(paste("Bags/predictionsSet",mar[i],".rds",sep=""))
# mar.risk<-cbind(mar.risk,risk)
# mar.risk<-rowSums(mar.risk)
# }
# apr.risk<-NULL
# for (i in 1:length(apr)){
```

```
# risk<-readRDS(paste("Bags/predictionsSet",apr[i],".rds",sep=""))
# apr.risk<-cbind(apr.risk,risk)
# apr.risk<-rowSums(apr.risk)
# }
# may.risk<-NULL
# for (i in 1:length(may)){
# risk<-readRDS(paste("Bags/predictionsSet",may[i],".rds",sep=""))
# may.risk<-cbind(may.risk,risk)
# may.risk<-rowSums(may.risk)
# }
# jun.risk<-NULL
# for (i in 1:length(jun)){
# risk<-readRDS(paste("Bags/predictionsSet",jun[i],".rds",sep=""))
# jun.risk<-cbind(jun.risk,risk)
# jun.risk<-rowSums(jun.risk)
# }
# jul.risk<-NULL
# for (i in 1:length(jul)){
# risk<-readRDS(paste("Bags/predictionsSet",jul[i],".rds",sep=""))
# jul.risk<-cbind(jul.risk,risk)
# jul.risk<-rowSums(jul.risk)
# }
# aug.risk<-NULL
# for (i in 1:length(aug)){
# risk<-readRDS(paste("Bags/predictionsSet",aug[i],".rds",sep=""))
# aug.risk<-cbind(aug.risk,risk)
# aug.risk<-rowSums(aug.risk)
# }
# sep.risk<-NULL
# for (i in 1:length(sep)){
# risk<-readRDS(paste("Bags/predictionsSet",sep[i],".rds",sep=""))
# sep.risk<-cbind(sep.risk,risk)
# sep.risk<-rowSums(sep.risk)
# }
# oct.risk<-NULL
# for (i in 1:length(oct)){
# risk<-readRDS(paste("Bags/predictionsSet",oct[i],".rds",sep=""))
# oct.risk<-cbind(oct.risk,risk)
# oct.risk<-rowSums(oct.risk)
# }
# nov.risk<-NULL
# for (i in 1:length(nov)){
# risk<-readRDS(paste("Bags/predictionsSet",nov[i],".rds",sep=""))
# nov.risk<-cbind(nov.risk,risk)
# nov.risk<-rowSums(nov.risk)
# }
# dec.risk<-NULL
# for (i in 1:length(dec)){
# risk<-readRDS(paste("Bags/predictionsSet",dec[i],".rds",sep=""))
# dec.risk<-cbind(dec.risk,risk)
# dec.risk<-rowSums(dec.risk)
# }
```
Get mean for each month across the 32 year period.
```{r}
# jan.mean<-jan.risk/length(jan)
# feb.mean<-feb.risk/length(feb)
# mar.mean<-mar.risk/length(mar)
# apr.mean<-apr.risk/length(apr)
# may.mean<-may.risk/length(may)
# jun.mean<-jun.risk/length(jun)
# jul.mean<-jul.risk/length(jul)
# aug.mean<-aug.risk/length(aug)
# sep.mean<-sep.risk/length(sep)
# oct.mean<-oct.risk/length(oct)
# nov.mean<-nov.risk/length(nov)
# dec.mean<-dec.risk/length(dec)
```
Take spillover intensity values for each month and turn them into a raster for each month.
```{r}
# jan.risk.r <-resampledRaster
# values(jan.risk.r)<-jan.mean
# feb.risk.r <-resampledRaster
# values(feb.risk.r)<-feb.mean
# mar.risk.r <-resampledRaster
# values(mar.risk.r)<-mar.mean
# apr.risk.r <-resampledRaster
# values(apr.risk.r)<-apr.mean
# may.risk.r <-resampledRaster
# values(may.risk.r)<-may.mean
# jun.risk.r <-resampledRaster
# values(jun.risk.r)<-jun.mean
# jul.risk.r <-resampledRaster
# values(jul.risk.r)<-jul.mean
# aug.risk.r <-resampledRaster
# values(aug.risk.r)<-aug.mean
# sep.risk.r <-resampledRaster
# values(sep.risk.r)<-sep.mean
```

````
# oct.risk.r <-resampledRaster
# values(oct.risk.r)<-oct.mean
# nov.risk.r <-resampledRaster
# values(nov.risk.r)<-nov.mean
# dec.risk.r <-resampledRaster
# values(dec.risk.r)<-dec.mean
```
Create a stack of Ebola spillover intensity by month.
```{r}
# risk.stack<-
stack(jan.risk.r,feb.risk.r,mar.risk.r,apr.risk.r,may.risk.r,jun.risk.r,jul.risk.r,aug.risk.r,sep.risk.r,oct.risk.r,nov
.risk.r,dec.risk.r)
# risk.brick<-brick(risk.stack)
# writeRaster(risk.brick,filename="risk.brick.grd",overwrite=T)
risk.brick<-brick("risk.brick.grd")
```
Create a dataframe of mean Ebola spillover by month and convert values to percentile.
```{r}
# mean.risk.df<-
cbind(jan.mean,feb.mean,mar.mean,apr.mean,may.mean,jun.mean,jul.mean,aug.mean,sep.mean,oct.mean,nov.mean,dec.mean)
# q<-ecdf(mean.risk.df)
# cum.risk.df<-q(mean.risk.df)
```
Take cumulative spillover intensity values for each month and turn them into a raster for each month.
```{r}
# jan.cum.r <-resampledRaster
# values(jan.cum.r)<-cum.risk.df[,1]
# feb.cum.r <-resampledRaster
# values(feb.cum.r)<-cum.risk.df[,2]
# mar.cum.r <-resampledRaster
# values(mar.cum.r)<-cum.risk.df[,3]
# apr.cum.r <-resampledRaster
# values(apr.cum.r)<-cum.risk.df[,4]
# may.cum.r <-resampledRaster
# values(may.cum.r)<-cum.risk.df[,5]
# jun.cum.r <-resampledRaster
# values(jun.cum.r)<-cum.risk.df[,6]
# jul.cum.r <-resampledRaster
# values(jul.cum.r)<-cum.risk.df[,7]
# aug.cum.r <-resampledRaster
# values(aug.cum.r)<-cum.risk.df[,8]
# sep.cum.r <-resampledRaster
# values(sep.cum.r)<-cum.risk.df[,9]
# oct.cum.r <-resampledRaster
# values(oct.cum.r)<-cum.risk.df[,10]
# nov.risk.r <-resampledRaster
# values(nov.cum.r)<-cum.risk.df[,11]
# dec.cum.r <-resampledRaster
# values(dec.cum.r)<-cum.risk.df[,12]
```
Create a stack of cumulative Ebola spillover intensity by month.
```{r}
# risk.stack2<-
stack(jan.cum.r,feb.cum.r,mar.cum.r,apr.cum.r,may.cum.r,jun.cum.r,jul.cum.r,aug.cum.r,sep.cum.r,oct.cum.r,nov.cum.r,dec
.cum.r)
# risk.brick2<-brick(risk.stack2)
# writeRaster(risk.brick2,filename="risk.brick2.grd",overwrite=T)
# risk.brick2<-brick("risk.brick2.grd")
```
Figure 1 - Map of known EVD events. Brown region indicates the focal region in Africa of annual rainfall > 600 mm.
Open circles indicate human spillovers, open triangles indicate infection/mortality in non-human primates or in other
mammals. Yellow, blue, green, magenta, and black indicate decade 1960-2010. Note that no Ebola spillovers are known
from the 1980s.
Get lat-long grid to overlay on map figures.
```{r}
latlonlines<-gridlines(risk.brick[[1]],easts = c(-10,0,10,20,30,40,50),norths=c(10,0,-10,-20,-30))
equator<-Line(cbind(c(-20,60),c(0,0)))
Lab.IO<-list("sp.text",c(48,-5),expression(atop(italic(Indian),italic(Ocean))),fontsize=list(text=6),font=2,
col="lightblue4")
Lab.AtO<-list("sp.text",c(-5,-15),expression(atop(italic(Atlantic),italic(Ocean))),fontsize=list(text=6),font=2,
col="lightblue4")
```
Get political map to overlay on map figures.
```{r}
countries<-getMap()
countries<-countries[!is.na(countries$continent),]
countries<-countries[countries$continent=="Africa",]
lakes<-shapefile("ne_10m_lakes.shp")
```
Separate human from non-human index points.
```{r}
index.h<-index.pts[ which(index.pts$human=="TRUE"), ]
index.nh<-index.pts[ which(index.pts$human=="FALSE"), ]
```
Set the coordinate reference to lat-long, and turn coordinates from index.pts into spatial points.
```{r}
index.h.pts<-SpatialPoints(index.h[,1:2],proj4string=crs("+proj=longlat +datum=WGS84 +no_defs +ellps=WGS84
+towgs84=0,0,0"))
````

index.nh.pts<-SpatialPoints(index.nh[,1:2],proj4string=crs("+proj=longlat +datum=WGS84 +no_defs +ellps=WGS84
+towgs84=0,0,0"))
```
Set colors for Ebola spillover locations by decade.
```{r}
dec<-
c("yellow","blue","blue","blue","blue","blue","blue","green","green","green","green","green","green","green","green","g
reen","green","green","dec2<-
c("green","green","green","green","magenta","magenta","magenta","magenta","magenta","magenta","magenta")
```
```{r}
spill.map<-spplot(mask,col.regions="tan",at=breaks,
colorkey=F,
xlab="Longitude",ylab="Latitude",
scales=list(x=list(draw=T,alternating=F),y=list(draw=T,alternating=F)),
par.settings=list(panel.background=list(col="lightblue1"),
layout.widths=list(axis.key.padding=1,ylab.right=4,ylab.left=4,right.padding=4),
layout.heights=list(top.padding=0.5,bottom.padding=0.5)),
axis.args=list(cex.axis=0.8),
sp.layout=list(list("sp.lines",latlonlines,col="lightblue3",lty=3,lwd=0.5,first=T),
list("sp.polygons",countries,fill="gray85",lwd=0),
list("sp.polygons",countries,lwd=0.6,first=F,col="white"),
list("sp.polygons",lakes,fill="lightblue1",lwd=0,col="lightblue1"),
list("sp.lines",equator,col="lightblue4",lty=1,lwd=1),
list("sp.points",index.h.pts,col=dec,pch=1,cex=2.2),
list("sp.points",index.nh.pts,col=dec2,pch=2,cex=2.2),
Lab.IO,Lab.AtO
)
)
print(spill.map)
```
Figure 2 - Spatio-temporal dynamics of EVD spillover intensity (the average density or expected number of points per
unit area and/or time) as percentile values by month for Africa varies seasonally. Modeled regions are restricted to
areas where rainfall > 600 mm annually. Lines at top and right depict the marginal probability by month (animated).
Set a color palette. Here going from cool to hot is most visually effective.
```{r}
risk.colors<-colorRampPalette(c("darkseagreen3","khaki","maroon"))
colors<-risk.colors(70)
breaks<-seq(0,1,length.out=70)
colorkey<-list(at=seq(0,1,0.02),labels=list(at=seq(0,1,0.1),cex=1.2),tick.number=11)
```
Make Ebola spillover intensity map figures by month.
```{r}
months<-
c("January","February","March","April","May","June","July","August","September","October","November","December")
risk.maps<-list()
for (i in 1:12){
risk.maps[[i]]<-spplot(risk.brick2[[i]],maxpixels=1000000,col.regions=colors,at=breaks,
colorkey=TRUE,
#main=list(label=months[i],cex=0.9,lines=1),
xlab="Longitude",ylab="Latitude",
scales=list(x=list(draw=T,alternating=F),y=list(draw=T,alternating=F)),#layout=c(3,4),
par.settings=list(panel.background=list(col="lightblue1"),
layout.widths=list(axis.key.padding=1,ylab.right=4,ylab.left=4,right.padding=4),
layout.heights=list(top.padding=0.5,bottom.padding=0.5)),
axis.args=list(cex.axis=0.6),
sp.layout=list(list("sp.lines",latlonlines,col="lightblue3",lty=3,lwd=0.5,first=T),
list("sp.polygons",countries,fill="gray85",lwd=0),
list("sp.polygons",countries,lwd=0.1,first=F,col="gray60"),
list("sp.lines",equator,col="lightblue4",lty=3,lwd=2),
list("sp.polygons",lakes,fill="lightblue1",lwd=0),
Lab.IO,Lab.AtO,
list("sp.text",c(-3,-32),months[i],fontsize=list(text=24),font=2,col="black")
)
)
}
names(risk.maps)<-months
panel.map<-c(risk.maps[["January"]],risk.maps[["February"]],risk.maps[["March"]],
risk.maps[["April"]],risk.maps[["May"]],risk.maps[["June"]],
risk.maps[["July"]],risk.maps[["August"]],risk.maps[["September"]],
risk.maps[["October"]],risk.maps[["November"]],risk.maps[["December"]],layout=c(3,4),merge.legends=FALSE)
```
Save to pdf.
```{r}
pdf(w=7.5,h=10,file="Ebola intensity.pdf",onefile=T)
print(panel.map)
grid.text("Predicted intensity",x=0.98,y=0.5,rot=90,gp=gpar(fontsize=12))
dev.off()
```
Repeat as an animated movie.
```{r}
library(animation)
gif<-function(){
for(i in 1:12)
{
risk.map<-spplot(risk.brick2[[i]],col.regions=colors,at=breaks,
colorkey=TRUE,
#main=list(label=months[i],cex=0.9,lines=1),

````
xlab="Longitude",ylab="Latitude",
scales=list(x=list(draw=T,alternating=F),y=list(draw=T,alternating=F)),#layout=c(3,4),
par.settings=list(panel.background=list(col="lightblue1"),
layout.widths=list(axis.key.padding=1,ylab.right=4,ylab.left=4,right.padding=4),
layout.heights=list(top.padding=0.5,bottom.padding=0.5)),
axis.args=list(cex.axis=0.6),
sp.layout=list(list("sp.lines",latlonlines,col="lightblue3",lty=3,lwd=0.5,first=T),
list("sp.polygons",countries,fill="gray85",lwd=0),
list("sp.polygons",countries,lwd=0.1,first=F,col="gray60"),
list("sp.lines",equator,col="lightblue4",lty=3,lwd=2),
Lab.IO,Lab.AtO,
list("sp.text",c(-3,-32),months[i],fontsize=list(text=12),font=2,col="black")
)
)
print(risk.map)
grid.text("Predicted spillover intensity",x=0.98,y=0.5,rot=90,gp=gpar(fontsize=12))
}
}
```
Save the animation of Ebola intensity maps of Africa by month.
```{r}
saveGIF(gif(), movie.name="SeasonalEbolaRisk.gif", interval=1, ani.width=1000, ani.height=800)
```
Create map of average Ebola spillover intensity.
```{r}
average.risk<-mean(risk.brick2)
average.map<-spplot(average.risk,col.regions=colors,at=breaks,
colorkey=TRUE,
xlab="Longitude",ylab="Latitude",
scales=list(x=list(draw=T,alternating=F),y=list(draw=T,alternating=F)),#layout=c(3,4),
par.settings=list(panel.background=list(col="lightblue1"),
layout.widths=list(axis.key.padding=1,ylab.right=4,ylab.left=4,right.padding=4),
layout.heights=list(top.padding=0.5,bottom.padding=0.5)),
axis.args=list(cex.axis=0.6),
sp.layout=list(list("sp.lines",latlonlines,col="lightblue3",lty=3,lwd=0.5,first=T),
list("sp.polygons",countries,fill="gray85",lwd=0),
list("sp.polygons",countries,lwd=0.1,first=F,col="gray60"),
list("sp.lines",equator,col="lightblue4",lty=3,lwd=2),
Lab.IO,Lab.AtO,
list("sp.text",c(-3,-32),"Annual
average",fontsize=list(text=10),font=2,col="black")
)
)
print(average.map)
```
Add animated lines at top and right depict the marginal intensity by month.
Mask risk.brick by mean percentile rank to capture > 0.5.
```{r}
rc1<-function(x){
ifelse(x>0.5,1,
ifelse(x<=0.5,NA,NA))}
risk.mask50<-calc(average.risk,fun=rc1)
risk.brick.m<-mask(risk.brick2,risk.mask50)
```
Set figure dimensions.
```{r}
theme.novpadding <-
list(layout.heights =
list(top.padding = 0,
main.key.padding = 0,
key.axis.padding = 0,
axis.xlab.padding = 0,
xlab.key.padding = 0,
key.sub.padding = 0,
bottom.padding = 0),
axis.line = list(col = 0),
clip =list(panel="off"),
layout.widths =
list(left.padding = 0,
key.ylab.padding = 0,
ylab.axis.padding = 0,
axis.key.padding = 0,
right.padding = 0))
```
Loop to get the averages and calculate row and column averages by month.
```{r}
col.ave<-NULL
smooth.col.ave<-NULL
row.ave<-NULL
smooth.row.ave<-NULL
for (i in 1:12){
col.ave<-rbind(col.ave,colSums(risk.brick.m[[i]],na.rm=T)/colSums(!is.na(risk.brick.m[[i]])))
smooth.col.ave<-cbind(smooth.col.ave,loess(col.ave[i,]~seq(1:length(col.ave[i,])),span=0.3)$fitted)
row.ave<-rbind(row.ave,rowSums(risk.brick.m[[i]],na.rm=T)/rowSums(!is.na(risk.brick.m[[i]])))
smooth.row.ave<-cbind(smooth.row.ave,loess(row.ave[i,]~seq(1:length(row.ave[i,])),span=0.3)$fitted)
}
col.max<-max(smooth.col.ave,na.rm=T)
row.max<-max(smooth.row.ave,na.rm=T)
````

~~~
```
Code lines representing monthly marginal averages by latitude and longitude to gray.
```{r}
top.plot<-list()
right.plot<-list()
for(i in 1:12){
top.plot[[i]]<-xyplot(smooth.col.ave[,i]~seq(1:length(col.ave[i,])),type="l",col="lightgray",
ylim=c(0.4,1),xlim=c(1,length(col.ave[i,])),
ylab="",xlab="",scales=list(x=list(draw=F),y=list(draw=F,tck=c(1,0))),
par.settings=list(theme.novpadding,axis.line = list(col = 0)),
panel=function(...){
lims <- current.panel.limits()
panel.xyplot(...)
})
right.plot[[i]]<-xyplot(smooth.row.ave[,i]~seq(1:length(row.ave[i,])),type="l",col="lightgray",
ylim=c(0.4,1),xlim=c(1,length(row.ave[i,])),
ylab="",xlab="",scales=list(x=list(draw=F),y=list(draw=F,tck=c(1,0))),
par.settings=list(theme.novpadding,axis.line = list(col = 0)),
panel=function(...){
lims <- current.panel.limits()
panel.xyplot(...)
})
}
```
Create loop to generate animated figure with line corresponding to month represented in red.
```{r}
gif2<-function(){
for(i in 1:12){
risk.map<-spplot(risk.brick2[[i]],maxpixls=1000000,col.regions=colors,at=breaks,
colorkey=list(space="bottom"),
#main=list(label=months[i],cex=0.9,lines=1),
xlab=list("Longitude",cex=1.4),
ylab=list("Latitude",cex=1.4),
scales=list(y=list(draw=T,alternating=F,at=c(-40,-30,-20,-10,0,10,20)),x=
(list(draw=T,at=c(-10,0,10,20,30,40,50)))),
axis.args=list(cex.axis=0.6),
#scales=list(x=list(draw=T,alternating=F),y=list(draw=T,alternating=F)),#layout=c(3,4),
par.settings=list(panel.background=list(col="lightblue1"),
layout.widths=list(axis.key.padding=0,ylab.right=0,ylab.left=0,right.padding=0,
top.padding = 0,
main.key.padding = 0,
axis.xlab.padding = 0,
xlab.key.padding = 0,
axis.ylab.padding = 0,
key.sub.padding = 0,
bottom.padding = 0),
layout.heights=list(top.padding = 0,
main.key.padding = 0,
key.axis.padding = 0,
axis.xlab.padding = 0,
xlab.key.padding = 4,
key.sub.padding = 4,
bottom.padding = 4,
axis.key.padding=4)),
axis.args=list(cex.axis=1.2),
sp.layout=list(list("sp.lines",latlonlines,col="lightblue3",lty=3,lwd=0.5,first=T),
list("sp.polygons",countries,fill="gray85",lwd=0),
list("sp.polygons",countries,lwd=1,first=F,col="gray50"),
list("sp.lines",equator,col="lightblue4",lty=3,lwd=2),
Lab.IO,Lab.AtO,
list("sp.polygons",lakes,fill="lightblue1",lwd=0),
list("sp.text",c(-3,-32),months[i],fontsize=list(text=24),font=2,col="black")
)
)
top.plot.current<-xyplot(smooth.col.ave[,i]~seq(1:length(col.ave[i,])),type="l",col="maroon",
ylim=c(0,1),xlim=c(1,length(col.ave[i,])),
ylab="",xlab="",scales=list(x=list(draw=F),y=list(draw=T,tck=c(1,0))),
par.settings=list(theme.novpadding,axis.line = list(col = 0)))
right.plot.current<-xyplot(smooth.row.ave[,i]~seq(1:length(row.ave[i,])),type="l",col="maroon",
ylim=c(0,1),xlim=c(1,length(row.ave[i,])),
ylab="",xlab="",scales=list(x=list(draw=F),y=list(draw=T,tck=c(1,0))),
par.settings=list(theme.novpadding,axis.line = list(col = 0)))
grid.newpage()
grid.text("Spatio-temporal dynamics of Ebola spillover intensity",x=0.45,y=0.96,gp=gpar(fontsize=28))
pushViewport(viewport(x = 0.0, y = 0,
height = 0.95, width = 1-0.15,
just = c("left", "bottom"),
name = "main"))
print(risk.map,newpage=F)
grid.text("Cumulative intensity (percentile)",x=0.53,y=0.15,gp=gpar(fontsize=16))
upViewport(1)
pushViewport(viewport(x = 0.05, y =.793,clip="inherit",
height = 0.15, width = .813,
just = c("left", "bottom"),
name = paste("t",i)))
print(top.plot[[1]]+top.plot[[2]]+top.plot[[3]]+top.plot[[4]]+top.plot[[5]]+top.plot[[6]]+top.plot[[7]]+top.plot[[8]]+t
op.plot[[9]]+top.plot[[# grid.text(label=c("0.2","0.4","0.6","0.8"),x = c(0,0,0,0),
# y = c(0.26,0.42,0.58,0.74),gp=gpar(fontsize=10))
~~~

upViewport(1)
pushViewport(viewport(x = 0.815, y =0.815,
height = 0.15, width = 0.59,angle=-90,
just = c("left", "bottom"),
name = paste("r",i)))
print(right.plot[[1]]+right.plot[[2]]+right.plot[[3]]+right.plot[[4]]+right.plot[[5]]+right.plot[[6]]+right.plot[[7]]+right.plot[[8]]+right.upViewport(1)
}
}
```
Save the animation of Ebola intensity maps of Africa by month with marginal intensity lines by latitude and longitude at top and side.
```{r}
saveGIF(gif2(),movie.name="Seasonal Ebola risk2.gif",interval=1, ani.width=1000, ani.height=1000)
```
Figure 3 - Phase graph showing the relationship between mean monthly rainfall and raw EVD spillover intensity, the average density or expected number of points per unit area and/or time, for known EVD locations in West and central Africa (closed circles) and locations in northeastern or southern Africa where model results indicate moderate to high EVD spillover intensity seasonally (open squares). Points are ordered by least to greatest monthly rainfall at each site.
Get lat-long coordinates of locations of interest.
```{r}
library(ggmap)
Meliandou<-geocode("meliandou guinea",source="google",output="latlon")
Yambuku<-geocode("yambuku democratic republic of congo",source="google",output="latlon")
Makouke<-geocode("makouke gabon",source="google",output="latlon")
Nzara<-geocode("nzara south sudan",source="google",output="latlon")
Lowero<-geocode("lowero uganda",source="google",output="latlon")
Tai_Forest<-geocode("tai cote d'ivoire",source="google",output="latlon")
Songo<-geocode("songo angola",source="google",output="latlon")
Mwinilunga<-geocode("mwinilunga zambia",source="google",output="latlon")
Masasi<-geocode("masasi tanzania",source="google",output="latlon")
Maxixe<-geocode("maxixe mozambique",source="google",output="latlon")
Jimma<-geocode("jimma ethiopia",source="google",output="latlon")
Lakato<-geocode("lakato madagascar",source="google",output="latlon")
```
List places, names, months.
```{r}
places<-rbind(Meliandou,Yambuku,Makouke,Nzara,Lowero,Tai_Forest,Songo,Mwinilunga,Masasi,Maxixe,Jimma,Lakato)
names<-c("Meliandou","Yambuku","Makouke","Nzara","Lowero","Tai
Forest","Songo","Mwinilunga","Masisi","Maxixe","Jimma","Lakato")
places<-cbind(places,names)
months<-c("Jan","Feb","Mar","Apr","May","Jun","Jul","Aug","Sep","Oct","Nov","Dec")
month.num<-c(1,2,3,4,5,6,7,8,9,10,11,12)
```
Extract average risk and average rainfall by month for each site.
```{r}
mel.risk<-extract(risk,places[1,1:2])
mel.rf<-extract(af.rf.brick,places[1,1:2])
mel<-as.data.frame(cbind(t(mel.risk),t(mel.rf)))
tai.risk<-extract(risk,places[6,1:2])
tai.rf<-extract(af.rf.brick,places[6,1:2])
tai<-as.data.frame(cbind(t(tai.risk),t(tai.rf)))
mak.risk<-extract(risk,places[3,1:2])
mak.rf<-extract(af.rf.brick,places[3,1:2])
mak<-as.data.frame(cbind(t(mak.risk),t(mak.rf)))
yam.risk<-extract(risk,places[2,1:2])
yam.rf<-extract(af.rf.brick,places[2,1:2])
yam<-as.data.frame(cbind(t(yam.risk),t(yam.rf)))
nza.risk<-extract(risk,places[4,1:2])
nza.rf<-extract(af.rf.brick,places[4,1:2])
nza<-as.data.frame(cbind(t(nza.risk),t(nza.rf)))
low.risk<-extract(risk,places[5,1:2])
low.rf<-extract(af.rf.brick,places[5,1:2])
low<-as.data.frame(cbind(t(low.risk),t(low.rf)))
son.risk<-extract(risk,places[7,1:2])
son.rf<-extract(af.rf.brick,places[7,1:2])
son<-as.data.frame(cbind(t(son.risk),t(son.rf)))
mwi.risk<-extract(risk,places[8,1:2])
mwi.rf<-extract(af.rf.brick,places[8,1:2])
mwi<-as.data.frame(cbind(t(mwi.risk),t(mwi.rf)))
mas.risk<-extract(risk,places[9,1:2])
mas.rf<-extract(af.rf.brick,places[9,1:2])
mas<-as.data.frame(cbind(t(mas.risk),t(mas.rf)))
max.risk<-extract(risk,places[10,1:2])
max.rf<-extract(af.rf.brick,places[10,1:2])
max<-as.data.frame(cbind(t(max.risk),t(max.rf)))
jim.risk<-extract(risk,places[11,1:2])
jim.rf<-extract(af.rf.brick,places[11,1:2])
jim<-as.data.frame(cbind(t(jim.risk),t(jim.rf)))
lak.risk<-extract(risk,places[12,1:2])
lak.rf<-extract(af.rf.brick,places[12,1:2])
lak<-as.data.frame(cbind(t(lak.risk),t(lak.rf)))
```
Make phase graph of average rainfall by month (x-axis) and average Ebola spillover intensity by month.
```{r}
par(mar=c(4,4,1,1))
mel <- mel[order(mel$V2),]

````
plot(mel$V2,mel$V1,type='o',col="red",pch=16,xlim=c(0,420),ylim=c(0.01,0.43),xlab="Rainfall (mm)",ylab="Ebola
spillover intensity")
text(380,0.2,"Meliandou, Guinea",col="red",cex=0.85)
par(new=TRUE)
tai<-tai[order(tai$V2),]
plot(tai$V2,tai$V1,type='o',col="blue",pch=16,xlim=c(0,420),ylim=c(0.01,0.43),xlab="",ylab="",xaxt='n',yaxt='n')
text(200,0.17,"Tai Forest, Cote d'Ivoire",col="blue",cex=0.85)
par(new=TRUE)
mak<-mak[order(mak$V2),]
plot(mak$V2,mak$V1,type='o',col="orange",pch=16,xlim=c(0,420),ylim=c(0.01,0.43),xlab="",ylab="",xaxt='n',yaxt='n')
text(380,0.135,"Makouke, Gabon",col="orange",cex=0.85)
par(new=TRUE)
yam<-yam[order(yam$V2),]
plot(yam$V2,yam$V1,type='o',col="darkgreen",pch=16,xlim=c(0,420),ylim=c(0.01,0.43),xlab="",ylab="",xaxt='n',yaxt='n')
text(260,0.22,"Yambuku, DRC",col="darkgreen",cex=0.85)
par(new=TRUE)
nza<-nza[order(nza$V2),]
plot(nza$V2,nza$V1,type='o',col="brown",pch=16,xlim=c(0,420),ylim=c(0.01,0.43),xlab="",ylab="",xaxt='n',yaxt='n')
text(270,0.083,"Nzara, South Sudan",col="brown",cex=0.85)
par(new=TRUE)
low<-low[order(low$V2),]
plot(low$V2,low$V1,type='o',col="purple",pch=16,xlim=c(0,420),ylim=c(0.01,0.43),xlab="",ylab="",xaxt='n',yaxt='n')
text(210,0.25,"Lowero, Uganda",col="purple",cex=0.85)
par(new=TRUE)
jim<-jim[order(jim$V2),]
plot(jim$V2,jim$V1,type='o',col="red",pch=22,xlim=c(0,420),ylim=c(0.01,0.43),xlab="",ylab="",xaxt='n',yaxt='n')
text(277,0.115,"Jimma, Ethiopia",col="red",cex=0.85)
par(new=TRUE)
son <- son[order(son$V2),]
plot(son$V2,son$V1,type='o',col="blue",pch=22,xlim=c(0,420),ylim=c(0.01,0.43),xlab="",ylab="",xaxt='n',yaxt='n')
text(240,0.10,"Songo, Angola",col="blue",cex=0.85)
par(new=TRUE)
mwi<-mwi[order(mwi$V2),]
plot(mwi$V2,mwi$V1,type='o',col="orange",pch=22,xlim=c(0,420),ylim=c(0.01,0.43),xlab="",ylab="",xaxt='n',yaxt='n')
text(325,0.03,"Mwinilunga, Zambia",col="orange",cex=0.85)
par(new=TRUE)
mas<-mas[order(mas$V2),]
plot(mas$V2,mas$V1,type='o',col="darkgreen",pch=22,xlim=c(0,420),ylim=c(0.01,0.43),xlab="",ylab="",xaxt='n',yaxt='n')
text(160,0.025,"Masisi, Tanzania",col="darkgreen",cex=0.85)
par(new=TRUE)
max<-max[order(max$V2),]
plot(max$V2,max$V1,type='o',col="purple",pch=22,xlim=c(0,420),ylim=c(0.01,0.43),xlab="",ylab="",xaxt='n',yaxt='n')
text(195,0.06,"Maxixe, Mozambique",col="purple",cex=0.85)
par(new=TRUE)
lak<-lak[order(lak$V2),]
plot(lak$V2,lak$V1,type='o',col="brown",pch=22,xlim=c(0,420),ylim=c(0.01,0.43),xlab="",ylab="",xaxt='n',yaxt='n')
text(340,0.36,"Lakato, Madagascar",col="brown",cex=0.85)
```
Bring in brick of monthly Ebola intensity predicted on the full data set, but using 1975 rather than 2015 population.
```{r}
#risk.brick75<-brick("risk.brick75.grd")
```
Get mean Ebola spillover intensity across months for 1975 and 2015.
```{r}
mean.risk75<-mean(risk.brick75)
mean.risk15<-mean(risk.brick)
```
Get difference in average annual risk from 1975 to 2015.
```{r}
risk.dif<-mean.risk2015-mean.risk75
```
Figure 4 - Change in annual spillover intensity, the average density or expected number of points per unit area
and/or time, between 1975 and 2015. Warm colors indicate increased spillover intensity, cool colors indicate
decreased spillover intensity.
Set color palette.
```{r}
risk.colors<-colorRampPalette(c("darkgreen","white","brown"))
colors<-risk.colors(8)
breaks<-c(-0.232,-0.15,-0.10,-0.05,0,0.05,0.10,0.15,0.224)
colorkey<-list(at=c(-0.15,-0.10,0,0.10,0.15),labels=list(at=c(-0.15,-0.10,0,0.10,0.15),cex=1.2),tick.number=5)
```
```{r}
risk.dif.map<-spplot(risk.dif,maxpixels=1000000,col.regions=colors,at=breaks,
colorkey=TRUE,
xlab=list("Longitude",cex=1.4),
ylab=list("Latitude",cex=1.4),
scales=list(x=list(draw=T,alternating=F),y=list(draw=T,alternating=F)),
par.settings=list(panel.background=list(col="lightblue1"),
layout.widths=list(axis.key.padding=1,ylab.right=4,ylab.left=4,right.padding=4),
layout.heights=list(top.padding=0.5,bottom.padding=0.5)),
axis.args=list(cex.axis=0.6),
sp.layout=list(list("sp.lines",latlonlines,col="lightblue3",lty=3,lwd=0.6,first=T),
list("sp.polygons",countries,fill="gray92",lwd=0),
list("sp.polygons",countries,lwd=0.5,first=F,col="gray60"),
list("sp.polygons",lakes,fill="lightblue1",lwd=0),
list("sp.lines",equator,col="lightblue4",lty=3,lwd=2),
Lab.IO,Lab.AtO)
)
````

print(risk.dif.map)
```
Get delta population by taking the log10 of the absolute difference between population counts per 25 km-2 grid cell 1975-2015.
```{r}
# pd.delta<-risk.dif
# values(pd.delta)<-as.matrix(log10(abs(10^pop15-10^pop75))*(10^pop15-10^pop75)/abs(10^pop15-10^pop75))
# writeRaster(pd.delta,"pd.delta.tif")
pd.delta<-raster("pd.delta.tif")
```
Figure 5 - Log10 (change in human population size) per 25 km-2 grid cell 1975-2015. Warm colors indicate increased population size, cool colors indicate population declines.
Set color palette.
```{r}
risk.colors<-colorRampPalette(c("darkgreen","white","brown"))
colors<-risk.colors(14)
breaks<-c(-7.474,-6,-5,-4,-3,-2,-1,0,1,2,3,4,5,6,7.66)
colorkey<-
list(at=c(-7.474,-6,-5,-4,-3,-2,-1,0,1,2,3,4,5,6,7.66),labels=list(at=c(-7.474,-6,-5,-4,-3,-2,-
1,0,1,2,3,4,5,6,7.66),cex=1.2),tick.number=```
Plot map of change in population 1975-2015.
```{r}
delta.pd.map<-spplot(pd.delta,maxpixels=1000000,col.regions=colors,at=breaks,
colorkey=TRUE,
xlab=list("Longitude",cex=1.4),
ylab=list("Latitude",cex=1.4),
scales=list(x=list(draw=T,alternating=F),y=list(draw=T,alternating=F)),
par.settings=list(panel.background=list(col="lightblue1"),
layout.widths=list(axis.key.padding=1,ylab.right=4,ylab.left=4,right.padding=4),
layout.heights=list(top.padding=0.5,bottom.padding=0.5)),
axis.args=list(cex.axis=0.6),
sp.layout=list(list("sp.lines",latlonlines,col="lightblue3",lty=3,lwd=0.6,first=T),
list("sp.polygons",countries,fill="gray92",lwd=0),
list("sp.polygons",countries,lwd=0.5,first=F,col="gray60"),
list("sp.polygons",lakes,fill="lightblue1",lwd=0),
list("sp.lines",equator,col="lightblue4",lty=3,lwd=2),
Lab.IO,Lab.AtO)
)
print(delta.pd.map)
```
Figure S1 - Population density and Ebola spillover locations for the masked region of Africa (> 600 mm annual rainfall) for each decade 1960-2010. Legend numbers represent human population ranges as powers of 10 per 25 km2. Red circles mark human spillovers, orange circlesnon-human primate and other mammal spillovers.
Set population breakpoints of log10 values per cell.
```{r}
breakpoints <- c(0,1,2,3,4,5,6)
```
Set colors at breakpoints used to bin population counts in bagged regression.
```{r}
colors <- c("gray88","gray88","gray76","gray55","gray55","gray55")
```
Get 44 final human and non-human Ebola points and separate by decade.
```{r}
index.h60<-index.h[ which(index.h$decade == 6), ]
index.h70<-index.h[ which(index.h$decade == 7), ]
index.nh70<-index.nh[ which(index.nh$decade == 7), ]
index.h90<-index.h[ which(index.h$decade == 9), ]
index.nh90<-index.nh[ which(index.nh$decade == 9), ]
index.h00<-index.h[ which(index.h$decade == 0), ]
index.nh00<-index.nh[ which(index.nh$decade == 0), ]
index.h10<-index.h[ which(index.h$decade == 1), ]
index.nh10<-index.nh[ which(index.nh$decade == 1), ]
```
Make trellis map figure of population density and Ebola spillovers.
```{r}
par(mfrow=c(3,2))
par(mar=c(0.5,0.5,0.5,0.5),bty="n")
logpop60<-raster("logpop60.tif")
plot(logpop60,breaks=breakpoints,col=colors,ylim=c(-35,15),xlim=c(-20,50),xaxt="n",yaxt="n",legend=T)
points(index.h60$x,index.h60$y,pch=1,col="red",cex=1.5)
title("1960", line = -0.5)
par(mar=c(0.5,0.5,0.5,0.5),bty="n")
logpop70<-raster("logpop70.tif")
plot(logpop70,breaks=breakpoints,col=colors,ylim=c(-35,15),xlim=c(-20,50),xaxt="n",yaxt="n",legend=FALSE)
points(index.nh70$x,index.nh70$y,pch=1,col="orange")
points(index.h70$x,index.h70$y,pch=1,col="red",cex=1.5)
title("1970", line = -0.5)
par(mar=c(0.5,0.5,0.5,0.5),bty="n")
logpop80<-subset(popbrick,1)
plot(logpop80,breaks=breakpoints,col=colors,ylim=c(-35,15),xlim=c(-20,50),xaxt="n",yaxt="n",legend=FALSE)
title("1980", line = -0.5)
par(mar=c(0.5,0.5,0.5,0.5),bty="n")
logpop90<-subset(popbrick,8)
plot(logpop90,breaks=breakpoints,col=colors,ylim=c(-35,15),xlim=c(-55,50),xaxt="n",yaxt="n",legend=FALSE)
points(index.nh90$x,index.nh90$y,pch=1,col="orange",cex=1.5)
points(index.h90$x,index.h90$y,pch=1,col="red",cex=1.5)
title("1990", line = -0.5)
par(mar=c(0.5,0.5,0.5,0.5),bty="n")
```

```
logpop00<-subset(popbrick,18)
plot(logpop00,breaks=breakpoints,col=colors,ylim=c(-35,15),xlim=c(-20,50),xaxt="n",yaxt="n",legend=FALSE)
points(index.nh00$x,index.nh00$y,pch=1,col="orange",cex=1.5)
points(index.h00$x,index.h00$y,pch=1,col="red",cex=1.5)
title("2000", line = -0.5)
par(mar=c(0.5,0.5,0.5,0.5),bty="n")
logpop10<-subset(popbrick,28)
plot(logpop10,breaks=breakpoints,col=colors,ylim=c(-35,15),xlim=c(-20,50),xaxt="n",yaxt="n",legend=FALSE)
points(index.nh10$x,index.nh10$y,pch=1,col="orange",cex=1.5)
points(index.h10$x,index.h10$y,pch=1,col="red",cex=1.5)
title("2010", line = -0.5)
```

Figure S2 - ROC curves showing performance of models resulting from analyses using all spillover points, predicted on 2/3 training data (top), and predicted on holdout 1/3 test data (middle). Bottom row shows performance of models resulting from analyses using human spillover points only predict on training data. Left and right columns compare results from 2 different sets of background points. Filled polygons represent the area under the ROC curve (AUC), red arcs are smoothed curves.

Training on test: all points.

```{r}
library(ROCR)
library(pROC)
test <- read.table("test.csv",header = T, sep=",",na.strings=c("na","NA"))
pred <- prediction(test[,1],test[,2])
perf <- performance(pred,"sens","spec")
plot(perf)
```

Training on training: human spillovers only.

```{r}
test.h<-read.table("test.h.csv",header = T, sep=",",na.strings=c("na","NA"))
pred2 <- prediction(test.h[,1],test.h[,2])
perf2 <- performance(pred2,"sens","spec")
plot(perf2)
```

Training on training: all points.

```{r}
test.a<-read.table("test.all.csv",header = T, sep=",",na.strings=c("na","NA"))
pred3 <- prediction(test.a[,1],test.a[,2])
perf3 <- performance(pred3,"sens","spec")
plot(perf3)
```

Create paired graphs.

```{r}
par(mfrow=c(3,2))
test.a<-read.table("test.all.csv",header = T, sep=",",na.strings=c("na","NA"))
rocobj3<-roc(test.a[,2],test.a[,1])
plot(rocobj3,col="blue",print.auc=T,main=" ",print.auc.cex=1.15,auc.polygon=T)
plot.roc(smooth(rocobj3), add=TRUE, col="red")
title("All Spillovers:", line=3,adj=1)
test.a2<-read.table("test.all2.csv",header = T, sep=",",na.strings=c("na","NA"))
rocobj4<-roc(test.a2[,2],test.a2[,1])
plot(rocobj4,col="blue",print.auc=T,main=" ",print.auc.cex=1.15,auc.polygon=T)
plot.roc(smooth(rocobj4), add=TRUE, col="red")
title("Training on Training", line=3,adj=0)
test <- read.table("test.csv",header = T, sep=",",na.strings=c("na","NA"))
rocobj<-roc(test[,2],test[,1])
plot(rocobj,col="blue",print.auc=T,main=" ",print.auc.cex=1.15,auc.polygon=T)
plot.roc(smooth(rocobj), add=TRUE, col="red")
title("All Spillovers:", line=3,adj=1)
test2 <- read.table("test2.csv",header = T, sep=",",na.strings=c("na","NA"))
rocobj5<-roc(test2[,2],test2[,1])
plot(rocobj5,col="blue",print.auc=T,main=" ",print.auc.cex=1.15,auc.polygon=T)
plot.roc(smooth(rocobj5), add=TRUE, col="red")
title("Training on Test", line=3,adj=0)
test.h<-read.table("test.h.csv",header = T, sep=",",na.strings=c("na","NA"))
rocobj2<-roc(test.h[,2],test.h[,1])
plot(rocobj2,col="blue",print.auc=T,main=" ",print.auc.cex=1.15,auc.polygon=T)
plot.roc(smooth(rocobj2), add=TRUE, col="red")
title("Human Spillovers:", line=3,adj=1)
test.h2<-read.table("test.h2.csv",header = T, sep=",",na.strings=c("na","NA"))
rocobj6<-roc(test.h2[,2],test.h2[,1])
plot(rocobj6,col="blue",print.auc=T,main=" ",print.auc.cex=1.15,auc.polygon=T)
plot.roc(smooth(rocobj6), add=TRUE, col="red")
title("Training on Training", line=3,adj=0)

```
